# Understanding Hepatopancreas-Associated Microbiota in the Supralittoral *Tylos ponticus* (Crustacea, Isopoda, Oniscidea): Insights from Next-Generation Sequencing Approaches

**DOI:** 10.1007/s00248-026-02785-4

**Published:** 2026-05-23

**Authors:** Domenico Davolos, Claudio Chimenti, Giulia Fassio, Valeria Russini, Andrea Lepri, Elisa Nocella

**Affiliations:** 1 Department of Biology and Biotechnologies “Charles Darwin”, Sapienza University of Rome, Rome, 00185 Italy; 2 Department of Technological Innovations and Safety of Plants, Products and Anthropic Settlements (DIT), INAIL, Via R. Ferruzzi 38/40, Research Area, Rome, 00143 Italy; 3 Istituto Zooprofilattico Sperimentale del Lazio e della Toscana “M. Aleandri”, via Appia Nuova, Rome, 1411, 00178 Italy; 4 Department of Biology and Evolution of Marine Organisms, Stazione Zoologica Anton Dohrn, Naples, 00198 Italy

**Keywords:** 16S metabarcoding, CAZymes, Holobiont, Isopods, Lignocellulose, Microbiota, Shotgun metagenomics, Whole genome sequencing

## Abstract

*Tylos* isopods, which are found exclusively in supralittoral beaches, play an important ecological role in the harsh sea-land interface contributing significantly to lignocellulose degradation. Herein, we investigated the hepatopancreatic microbiota in the oniscidean isopod *Tylos ponticus* Grebnitzky, 1874 from an Italian supralittoral zone characterized by the accumulation of beached leaves from the seagrass *Posidonia oceanica*. To characterize this *Tylos*–microbe system, we combined three Next Generation Sequencing techniques: 16S rRNA gene metabarcoding, whole-genome sequencing of cultured hepatopancreatic bacteria and shotgun metagenomic sequencing of uncultured bacterial communities. Comparative analyses revealed that some bacterial taxa were associated with the hepatopancreas of *T. ponticus* but were also detected in the supralittoral sandy beach where the detritivores *Tylos* live. However, distinct components of the microbial community may be adapted within the hepatopancreas. Moreover, the assembled and annotated genomes of hepatopancreatic bacteria allowed us to identify genes encoding lignocellulose-degrading CAZymes for a better understanding of the role of symbionts in aiding lignocellulose degradation. Finally, our shotgun sequencing data confirmed the presence of an uncultured *Candidatus* Hepatoplasma (Mollicutes) in the hepatopancreas of *T. ponticus*, with the provisional taxonomic assignment as *Candidatus* Hepatoplasma cf. vulgare Tp. We compared this data with recently reported metagenome-assembled genomes of uncultured Hepatoplasmataceae members from isopods, including *Candidatus* Tyloplasma litorale identified from the semiterrestrial isopod *Tylos granuliferus*, *Candidatus* Hepatoplasma vulgare from the terrestrial isopod *Armadillidium vulgare*, and *Candidatus* Hepatoplasma scabrum from the terrestrial isopod *Porcellio scaber*. In such a scenario, a deeper understanding of halophilic bacteria in the supralittoral zone also has broad relevance to applied research, particularly to the biotechnological sector related to marine biomass conversion and plastic degradation.

**Supplementary Information:**

The online version contains supplementary material available at 10.1007/s00248-026-02785-4.

## Introduction

Oniscidean isopods (Malacostraca, Peracarida) are the only group of crustaceans predominantly composed of terrestrial species and represent the most successful crustacean lineage in the colonization of terrestrial habitats [1, 2]. The ecological range of oniscideans extends from supralittoral zones to arid landscapes, encompassing environments from sea level to high-altitude regions, as well as subterranean habitats such as caves [3]. Among the oniscidean lineages, Tylidae includes species of genus *Tylos* inhabiting the supralittoral zone, which represents the transition between sea and land, play a crucial role in coastal ecosystems by actively participating in the decomposition of organic matter, such as the beached leaves of the seagrass *Posidonia oceanica* (*Posidonia* banquette) [4]. This environment is characterized by extreme conditions, including high salinity, intense solar exposure, and limited freshwater availability, which necessitate specific adaptive strategies by the organisms residing there [5]. A key aspect of the ability of oniscidean isopods to inhabit the supralittoral zone lies in their relationship with symbiotic microbiomes, which perform essential roles in organic material digestion, detoxification of harmful compounds, and resistance to environmental stressors. In particular, gut microbiomes contribute to the metabolism of complex substances, such as cellulose found in *P. oceanica* leaves, and influence the fitness and survival of isopod populations in these coastal habitats.

**Fig. 1:**
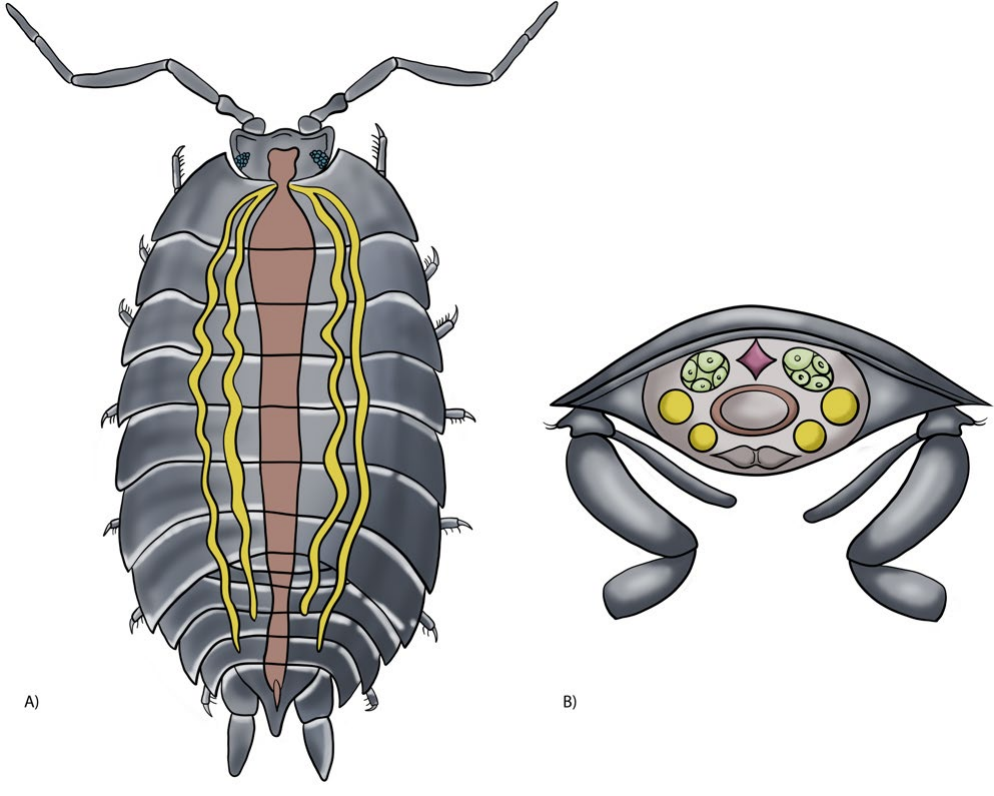
Generalized scheme (A) and transverse section (B) of the digestive tract of oniscidean isopods. Yellow=hepatopancreas, Brown=digestive system, Green=ovaries, Red=aorta

In *Tylos ponticus* Grebnitzky, 1874, like other oniscidean isopods, the ectodermal digestive tract is composed of the foregut and the hindgut, with two pairs of lateral midgut gland tubules (lateral caeca) that form the hepatopancreas (Fig. 1). These four endodermal hepatopancreatic tubules are connected to the digestive tract at the junction of the foregut and hindgut [6]. The fine vegetable materials extracted from the stomach (foregut chambers) are normally filtered through the filter systems (the paired primary and secondary filter covered with brush-like structures and underlain by grooves) and then conveyed to hepatopancreatic tubules, which are lined by a pseudostratified epithelium consisting of different cell types, while the larger vegetable particles are carried towards the gut lumen [6].

Recent studies have highlighted that once plant biomass is ingested, digestive enzymes (Carbohydrate-Active enZymes, called CAZymes) degrade lignin, cellulose and hemicellulose [7]. Like other terrestrial oniscidean isopods, in *T. ponticus* the lignocellulose degradation likely occurs at the holobiont level (*Tylos*-microbe system), with decomposition of lignocellulose by microbiomes likely representing an essential process. In particular, the hepatopancreas microbial symbionts may be essential for feeding on marine vegetable materials available in the sea-land interface where the supralittoral detritivores *Tylos* live. Indeed, it is becoming increasingly evident that oniscidean isopods degrading lignocellulose use a combination of endogenous and microbial enzymes (e.g., by means of their hepatopancreas symbionts), as recently demonstrated in the terrestrial *Armadillidium vulgare* (Latreille, 1804) [8]. Specifically, the filtrate via the atrium of the midgut glands is ultimately conveyed to the hepatopancreatic lumen in which endogenous and microbial CAZymes are produced and secreted for lignocellulose digestion.

Although the microbiome of *Tylos* has begun to be investigated using 16S rRNA gene sequencing [4], a microbial symbiotic association has been hypothesized to underlie the hepatopancreatic bacterial communities of *T. ponticus*. A comprehensive understanding of the genomes of bacteria isolated from the hepatopancreas, particularly with respect to their functional potential, such as cellulolytic capabilities, is still lacking. Moreover, representative metagenomic data from uncultured bacterial taxa inhabiting the hepatopancreas of *T. ponticus* remain to be explored.

Our work was therefore designed to investigate the symbiotic microbiomes of the oniscidean isopod *Tylos ponticus*, inhabiting the Mediterranean supralittoral zone, an area characterized by the accumulation of beached leaves from the seagrass *Posidonia oceanica*. In this study, we aim to address three main objectives. First, in light of the complex taxonomy of *Tylos* isopods, we assessed whether the collected specimens could be reliably assigned to *T. ponticus*. Second, we conducted a histological investigation of the hepatopancreas of *T. ponticus*, given the central role of this organ in oniscidean isopods [8]. Third, we characterized the microbiota of the hepatopancreas of *T. ponticus* by exploiting the scalability and flexibility of next-generation sequencing (NGS) technologies to obtain high-resolution insights into the complex *Tylos*–microbe system. To this end, we employed three complementary NGS approaches: (i) comparative metabarcoding analysis of *T. ponticus* hepatopancreas, hindguts, and sand sample, targeting hypervariable regions of the bacterial 16S rRNA gene, (ii) whole-genome sequencing (WGS) of bacteria isolated and cultured from the hepatopancreas, and (iii) shotgun metagenomic sequencing of uncultured hepatopancreatic bacterial communities.

In particular, we examined the hepatopancreas samples using both morphological and molecular approaches, together with the associated microbial communities from the supralittoral habitat inhabited by *T. ponticus*. Two guts from the same individuals were also analysed using the same approach, allowing a direct comparison of the microbiota associated with different internal organs within the same host. We also assembled through whole genome sequencing and annotated genomes of bacteria isolated and cultured from the hepatopancreas of *T. ponticus* to improve our understanding of their role in aiding lignocellulose degradation. Finally, we carried out a shot-gun sequencing to study the metagenome-assembled genomes of uncultured Hepatoplasmataceae members in the Mollicutes from the hepatopancreas of *T. ponticus*. We compared *Candidatus* Hepatoplasma sp. associated with *T. ponticus* with recently reported metagenome-assembled genomes (MAGs) of uncultured members of the family Hepatoplasmataceae from isopods. These included *Candidatus* Tyloplasma litorale identified in the semiterrestrial isopod *Tylos granuliferus* Budde-Lund, 1885, *Candidatus* Hepatoplasma vulgare from the terrestrial isopod *Armadillidium vulgare*, and *Candidatus* Hepatoplasma scabrum from *Porcellio scaber* Latreille, 1804 [9].

## Materials and Methods

### Isopod and Environmental Sampling

**Fig. 2:**
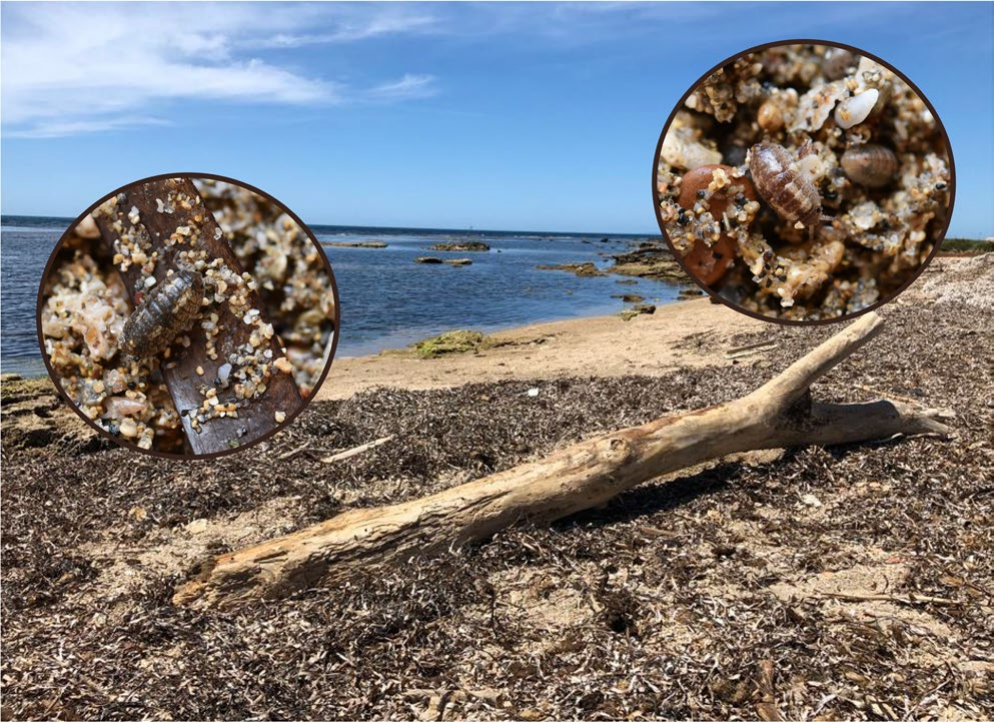
*Tylos ponticus* (zoomed in circles) collected from a supralittoral banquette composed of beached leaf litter of the seagrass *Posidonia oceanica*, Civitavecchia, Lazio, Italy

*Tylos ponticus* specimens were collected by hand from a supralittoral *Posidonia* banquette composed of leaf litter from the seagrass *Posidonia oceanica* (Fig. 2), located on a beach along the central Tyrrhenian Sea (Sant’Agostino, Civitavecchia, Italy; 42° 9′ 33″ N, 11° 44′ 4″ E).

Most Isopods samples were preserved in molecular grade ethanol upon collection, while others were fixed in Bouin’s fluid for histological analysis following the standard protocol described by [10]. Simultaneously, environmental samples of sand were collected and in the same area as the crustacean specimens and stored in sterile 10 mL tubes. Morphological identification was carried out under a Wild-M6 microscope. The hindgut, hepatopancreas, and pereopods of *T. ponticus* were excised using sterile forceps and immediately processed for DNA extraction (see below).

## DNA Extraction and Molecular Analyses

All samples were processed at the Molecular Systematic Laboratory, Department of Biology and Biotechnologies “Charles Darwin”, Sapienza University of Rome, Italy. Total genomic DNA was extracted from the hindgut, from the hepatopancreas, from the pereopods (for phylogenetic analysis, see below) and from the sand using Zymobiomics DNA extraction kit following the manufacturer’s protocol.

Considering the reported issues of phenotypic plasticity in genus *Tylos* [5], we tested that all the collected specimens (Tyl-1‒5) belonged to the same species, employing an integrative taxonomy approach. The 652 bp barcode region of the mitochondrial cytochrome oxidase subunit 1, *cox1*, was amplified following the protocols outlined by [11, 12]. In addition, a 319 bp region of the H3 histone gene was amplified following the PCR conditions of [13]. Polymerase chain reactions were performed in 25 µl reaction volume following [11]. The resulting PCR products were purified using ExoSAP-IT, and both strands were sequenced at BMR Genomics (Padova, Italy). Sequences were aligned using the Geneious following [14]. Phylogenetic analyses were performed on the combined gene datasets (*cox1* partitioned by codon and H3) with Bayesian inference (BI) approach. JModeltest v.2 [15] was used to choose for each gene the best fitting nucleotide substitution model (*cox1* codon 1st = HKY + F + I, 2nd = HKY+F, 3rd = TNe+G4; H3 = JC). Analyses were performed using MrBayes v.3 (10^7^ generation, 25% burn-in, other parameters as default [16]) on CIPRES Science Gateway Portal [17]. MCMC convergence was checked with Tracer v.1.7 [18]. Sequences were deposited in GenBank (See Suppl. Table S1).

## Histological Analysis

Isopods sample fixation was carried out for 24 h at room temperature to ensure optimal preservation of cellular and subcellular structures. After fixation, the samples were washed thoroughly in running tap water to remove excess picric acid and prevent artifacts during subsequent processing. The specimens were kept in 70% ethanol for 1 h and then in 80% ethanol for 2 days to clear the samples of Bouin’s solution. After dehydration in graded concentrations of ethanol at 4 °C, the specimens were cleared in histolemon (three times, each 10 min) and finally embedded in paraffin wax at 60 °C (including an overnight step, for better wax penetration). Transverse serial sections, each 6 μm thick, were obtained using a rotary microtome (Leica RM2235, Germany) to ensure consistency and precision across sections.

The sections were mounted on glass slides pre-coated with albumin-glycerol adhesive to prevent detachment during staining procedures. Staining was carried out using haematoxylin-eosin (H&E) as described by [6], to highlight cellular and subcellular details. H&E staining was used to differentiate nuclei and cytoplasm, providing general histological context, while Giemsa staining was employed for specific visualization of chromatin and cytoplasmic details, particularly in cells of hematopoietic or microbial origin. Histological sections were examined using a light microscope (Nikon Eclipse E400) equipped with a digital camera for photomicrography (Zeiss Axiocam 208 color).

### 16S rRNA Metabarcoding Analysis

For metabarcoding analysis of five hepatopancreas (Tyl-1H–Tyl-5H) and two hindguts (Tyl-1G–Tyl-2G) of *Tylos ponticus* and a sample of sand (S-1), the universal primers Pro341F: 5′-CCTACGGGNBGCASCAG-3′ and Pro805R: Rev 5′-GACTACNVGGGTATCTAATCC-3′ [19] were applied to capture the V3-V4 region of 16S rDNA. The PCR reaction system was composed of 5 µL of buffer, 0.25 µL of PCRBIO HIFI DNA Polymerase (1000 Units), 2 µL (2.5 mM) of dNTPs, 0.5 µL (10 µM) of each forward and reverse primer, 5 µL of DNA Template (10 ng/µl), and 13.75 µL of ddH2O. The PCR program was set as follows: 95 °C for 3 min; 25 cycles of 95 °C for 30 s, 55 °C for 30 s, 72 °C for 30 s; and 72 °C for 5 min.

The sequencing of 16 S V3–V4 fragments was performed on an Illumina MiSeq platform (Illumina, San Diego, CA, USA) and DNA library was prepared using Nextera XT kit (Illumina, San Diego, CA, USA). For indexing reaction by primer Nextera XT Index Kit v2, the PCR reaction system was composed of 5 µL of buffer, 0.25 µL of PCRBIO HIFI DNA Polymerase (1000 Units), 2.5 µL Index 1 and Index2, 5 µL of DNA, and 9.75 µL of ddH2O.The PCR program was set as follows: 95 °C for 3 min; 8 cycles of 95 °C for 30 s, 55 °C for 30 s, 72 °C for 30 s; and 72 °C for 5 min. Libraries were validated with Bioanalyzer 2100 (Agilent Technologies, Palo Alto, CA, USA) and were then sequenced using the MiSeq at the Bio-Fab research srl (Rome, Italy). DNA template and library were quantified with a Qubit 2.0 Fluorometer (Invitrogen). All the procedures were performed according to the manufacturer’s instructions.

For quality control analysis, raw sequence data generated on the Illumina platform in FASTQ format were assessed using FastQC version 0.11.9 (https://kbase.us/applist/apps/kb_fastqc/runFastQC/release; UK, http://www.bioinformatics.babraham.ac.uk/projects/fastqc/, accessed on 2026).

The QIIME 2 v2019.10 pipeline (https://qiime2.org/; [20]) was used for the 16S rDNA sequence analysis using amplicon sequence variant (ASV). Sequence denoising, filtering, and trimming were performed using the DADA2 plugin within QIIME 2. Reads shorter than 150 bp were discarded, and default parameters were applied for quality filtering and error correction. Next, we use the ASV table in the feature-classifier step to generate taxonomy assignment. The sequencing reads were assigned to taxonomic identities using the Silva 138 release database (https://www.arb-silva.de/documentation/release-138/ accessed on April 2023). For each sample, a matrix of ASV abundances with significant taxonomic identification was assembled using the taxonomic assignments and the ASV map. Diversity analyses were not performed at the ASV species level due to the limited taxonomic resolution of 16S rRNA gene metabarcoding, which may lead to unreliable species-level assignments. For this reason, analyses were conducted at higher taxonomic levels (genus and family), which provide more robust and interpretable ecological patterns.

Taxonomic bar plots were generated in RStudio (v2023.12.1, Build 402) based on ASVs at both the family and genus levels. Relative abundance of bacterial taxa was calculated using the packages “dplyr” and “tidyr”. For each sample, counts were normalized to percentages, and taxa representing less than 2% relative abundance were set to zero. The remaining taxa counts were subsequently re-normalized to sum to 100% within each sample. Data were visualized as stacked bar plots using the package “ggplot2”.

Alpha rarefaction curves (created using the plugin diversity alpha-rarefaction in QIIME2) were utilised to assess bacterial communities’ diversity across different sequencing depths. Rarefaction was performed by random subsampling of sequencing reads without replacement at increasing depths, and diversity metrics were computed at each level. This method enables the estimation of how bacterial community diversity changes according to sequencing efforts.

The alpha diversity for each sample was calculated with QIIME2 on relative abundance of the detected families and genera using three diversity indexes: Shannon, Simpson and Chao1. The beta diversity for each sample was evaluated with QIIME2 using Bray-Curtis distances at the family and genus levels.

 Principal Coordinates Analysis (PCoA) of beta diversity was performed on the ASV datasets, based on Bray-Curtis distances of the microbial composition at the family and genus and levels. The Principal Coordinates Analysis (PCoA) was performed using RStudio (2023.12.1 Build 402). The “vegan” package was used to perform ecological and multivariate statistical analyses; the cmdscale function was employed for PCoA computation, while the “plotly” package [21] was used to facilitate interactive 3D visualization of the PCoA results, allowing for an enhanced graphical representation. Additionally, “dplyr” [22] and “RColorBrewer” [23] packages have been used for efficient data manipulation and to provide a distinct colour palette to the sample in the 3D plot, respectively. Identification of the bacterial genera shared between hepatopancreas and hindgut samples was performed following [24], allowing the generation of a common bacterial genus abundance heatmap, employing the “ComplexHeatmap” package, where rows represent the analysed samples and columns indicate the relative frequency of shared bacterial ASVs identified as genera (see Table S2).

The microbial DNA sequencing data obtained in the present study were submitted to the Sequence Read Archive (SRA) at GenBank database (National Center for Biotechnology Information, NCBI, https://www.ncbi.nlm.nih.gov/, accessed on January 2026): bioproject PRJNA860326.

## Whole Genome Sequencing of Bacterial Strains

The hepatopancreas of an adult *Tylos ponticus* specimen (Tyl-2H) was carefully dissected using sterile forceps, placed in sterile 0.2 mL tubes containing sterile water, and homogenized by vortexing with 0.2 mL of sterile seawater. Aliquots of 0.1 mL from each homogenate were spread onto Nutrient Agar (NA) plates. The plates were then incubated at 30 °C in the dark for 48 h. Following incubation, cultivable bacteria displaying distinct colors, growth rates, and colony morphologies were selected. A single representative isolate from each morphotype was subcultured on fresh NA plates to obtain pure strains through successive passages. The purified strains were subsequently centrifuged at 10,000 × g for 90 s, and the bacterial pellets were transferred to sterile 2 mL tubes. Genomic DNA was extracted using the QIAamp DNA Microbiome Kit (Qiagen, Hilden, Germany), following the manufacturer’s protocol, and DNA concentrations were measured spectrophotometrically with a Nanodrop 2000 (Thermo Scientific).

Before performing genome-based analyses, the purified bacterial strains were preliminarily identified based on their 16S rRNA gene sequences. Selected strains, belonging to bacterial families known for their role in the degradation of plant polysaccharides in marine environments, were then subjected to WGS. The bacterial strains detected in the bacterial community associated with the hepatopancreas of *T. ponticus* were maintained in 30% glycerol at − 20 °C for long term storage.

Genomic DNA from *Vreelandella venusta* strain H3 was used for Illumina library preparation with the Nextera XT kit. DNA quality and concentration were assessed via Qubit 2.0, and libraries validated with a Bioanalyzer 2100. Sequencing was performed on an Illumina MiSeq platform (2 × 300 bp paired-end, dual indexing) at Bio-Fab Research srl (Rome, Italy). Raw reads quality was checked with FastQC v.0.11.9, and adapters removed using Trimmomatic v.0.38 [25]. Filtered reads were assembled *de novo* and annotated using Proksee [26] and Prokka [27]. Average Nucleotide Identity (ANI) comparisons were performed with JSpeciesWS (ANIb; [28]). Genome assembly metrics were obtained via QUAST v.4.5 [29]; tRNA genes predicted with tRNAscan-SE v.2.0 [30]. Genome completeness and annotation quality were assessed with BUSCO; gene prediction used AUGUSTUS [31] with *E. coli* K12 as training. Functional annotation was done with PANNZER2 [32], assigning Gene Ontology terms for molecular function, biological processes, and localization. Protein IDs (ProtID) and GenBank accession numbers (GbID) were used as gene identifiers. Carbohydrate-active enzymes (CAZymes) were identified via dbCAN3 [33], integrating HMMER, DIAMOND, and Hotpep searches with stringent thresholds. Secondary metabolite biosynthetic gene clusters (BGCs) were detected and characterized using antiSMASH v.8.0 [34] with relaxed stringency and compared against the MIBiG database. Synteny and homology of BGCs were visualized using [35] with GenBank files generated by antiSMASH.

## Shotgun Sequencing

The genomic DNA extracted from the hepatopancreas of samples Tyl-2H and Tyl-4H was used for Illumina library preparation. DNA quality and concentration were measured with a Qubit 2.0 Fluorometer (Invitrogen). Paired‑end libraries were prepared using the Nextera XT kit (Illumina, San Diego, CA, USA) and quantified on the Qubit 2.0, then validated on a Bioanalyzer 2100 (Agilent Technologies, Palo Alto, CA, USA) and sequenced using NovaSeq 6000 instrument (Illumina, San Diego, CA, USA) with a 2 × 150 paired end run with double indexing of the library at the Bio-Fab research srl (Rome, Italy). Quality assessment was calculated using Quast v5.3.0 [29].

Shotgun metagenomic reads (FASTQ) were processed via Galaxy (https://usegalaxy.eu/ accessed on 2024): quality‑checked with FastQC v0.11.9 (https://kbase.us/applist/apps/kb_fastqc/runFastQC/release; http://www.bioinformatics.babraham.ac.uk/projects/fastqc/*)*, adapter‑trimmed with Trimmomatic [25], taxonomically classified by Kraken2 v2.1.3 [36], and visualized with Krona [37]. For genome‑resolved metagenomics targeting *Candidatus Hepatoplasma* (family *Candidatus* Hepatoplasmataceae; [38, 39], trimmed reads were de novo assembled with MEGAHIT [40] and SPAdes v4.2.0 [27, 41], polished with Pilon [42], and binned with MetaBAT2 [43]. Draft contigs were annotated using Proksee [26], Prokka [27] and Pharokka [44]; orthologous clusters and phylogenomic relationships were inferred with OrthoVenn3 [45]. Functional annotation was performed with PANNZER2 [32], and carbohydrate‑active enzymes were identified via dbCAN3—HMMER dbCAN (E‑value < 1e‑15, coverage > 0.35), DIAMOND CAZy (E‑value < 1e‑102) and HMMER dbCAN‑sub (E‑value<1e‑15, coverage > 0.35), referencing CAZy (http://www.cazy.org, accessed 1 May 2025) [33]. Secondary metabolite biosynthetic gene clusters were detected and characterized with antiSMASH v8.0 using signature profile HMMs [34]. A phylogenomic tree based on highly conserved single-copy genes was constructed using OrthoVenn3 to infer evolutionary relationships within the *Candidatus Hepatoplasmataceae* family, applying a maximum-likelihood approach under the JTT + CAT model.

## Results

### Phylogenetic Analysis

**Fig. 3:**
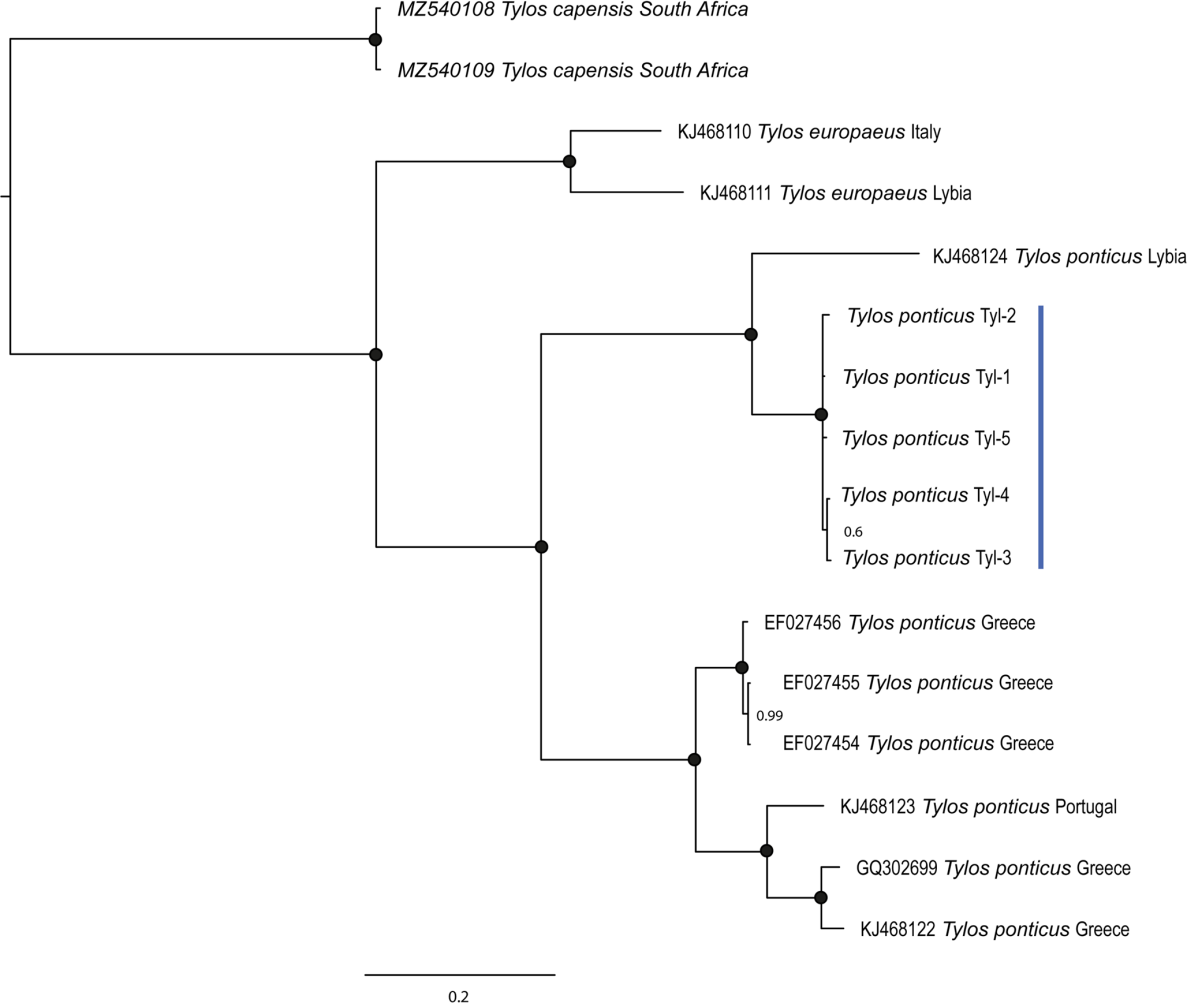
Phylogenetic relationships of the species *Tylos ponticus* (Bayesian Inference tree based on the combined dataset). Numbers at nodes indicate branch support values (PP). Black circles at nodes indicate maximum support (PP=1). The blue bar indicates sample sequences generated in this study

The molecular dataset was composed of 21 sequences (16 *cox1* and five H3) belonging to 16 individuals. The final alignment comprises 652 bp of *cox1* and 319 of H3. Among them, ten were generated in this study, while 11 *cox1* were obtained from the GenBank NCBI dataset, including two outgroup species: *Tylos europeus* Arcangeli, 1938, and *Tylos capensis* Krauss, 1843. Within the BI combined tree (*cox1* + H3, Fig. 3), the monophyly of *T. ponticus* was strongly supported with a Posterior Probability (PP) =1. Moreover, all the five samples sequenced in this work (Tyl-1‒5), morphologically ascribed to the same species, grouped within a strongly supported single clade (PP=1). However, the Italian samples, along with the Libyan one, appear to form a distinct clade separate from the remaining *T. ponticus* specimens from Greece and Portugal (PP=1).

## Histologically Investigation

**Fig. 4:**
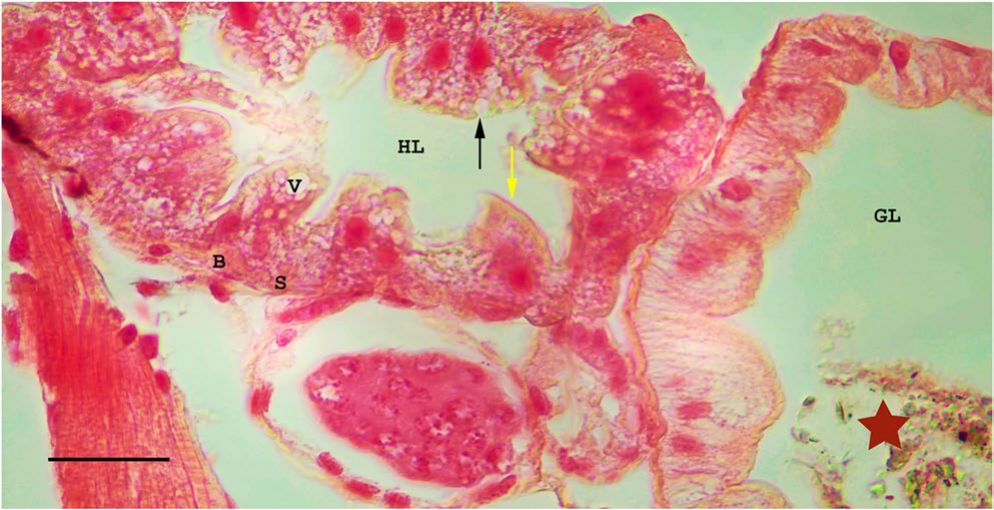
Cross section of one of the four hepatopancreatic caeca of *Tylos ponticus*, showing two epithelial cell types: dome-shaped B cells and smaller S cells, which alternate along the epithelium. Black arrows indicate vacuolization and secretory activity in B cells, while yellow arrows highlight the apical brush border. Fine plant-derived material in the gut lumen is marked by a red star. HL=hepatopancreatic lumen; GL=gut lumen; V=vacuole. Scale bar: 50 μm.

The two pairs of hepatopancreatic caeca of *Tylos ponticus* consist of tubular structures lined by a single layer of pseudostratified epithelial cells supported by a thin basal lamina (Fig. 4). Histological observations revealed that the pseudostratified epithelium of each tubule is composed of two main cell types, as previously described in other isopods (e.g. [6]) B cells (basal cells) and S cells (small cells), which were clearly distinguishable in the hepatopancreas of *T. ponticus*. B cells were dome-shaped, characterized by a vacuolated cytoplasm and a prominent apical brush border. These cells displayed marked vacuolization and secretory activity, often protruding apically into the hepatopancreatic lumen. In contrast, S cells were smaller and more compact, alternating with B cells along the epithelium and exhibiting a densely granular cytoplasm (Fig. 4). Fine plant-derived material was frequently observed within the gut lumen of *T. ponticus* (Fig. 4). 

### Bacterial Community Analysis by 16S rRNA Metabarcoding

About three million reads were correctly merged, and 2,704,460 reads (88%) were retained for subsequent analyses, with an average 450,000 reads per sample. Consensus data from the different hypervariable regions of the 16S rRNA gene were used for the analysis of bacteria from hepatopancreas and gut tissue and environmental samples at each taxonomic level. Rarefaction curves almost plateaued with saturation values (Supplementary Figure S1), indicating that the metabarcoding sequencing identified most of the bacterial taxa present in each sample.

**Fig. 5:**
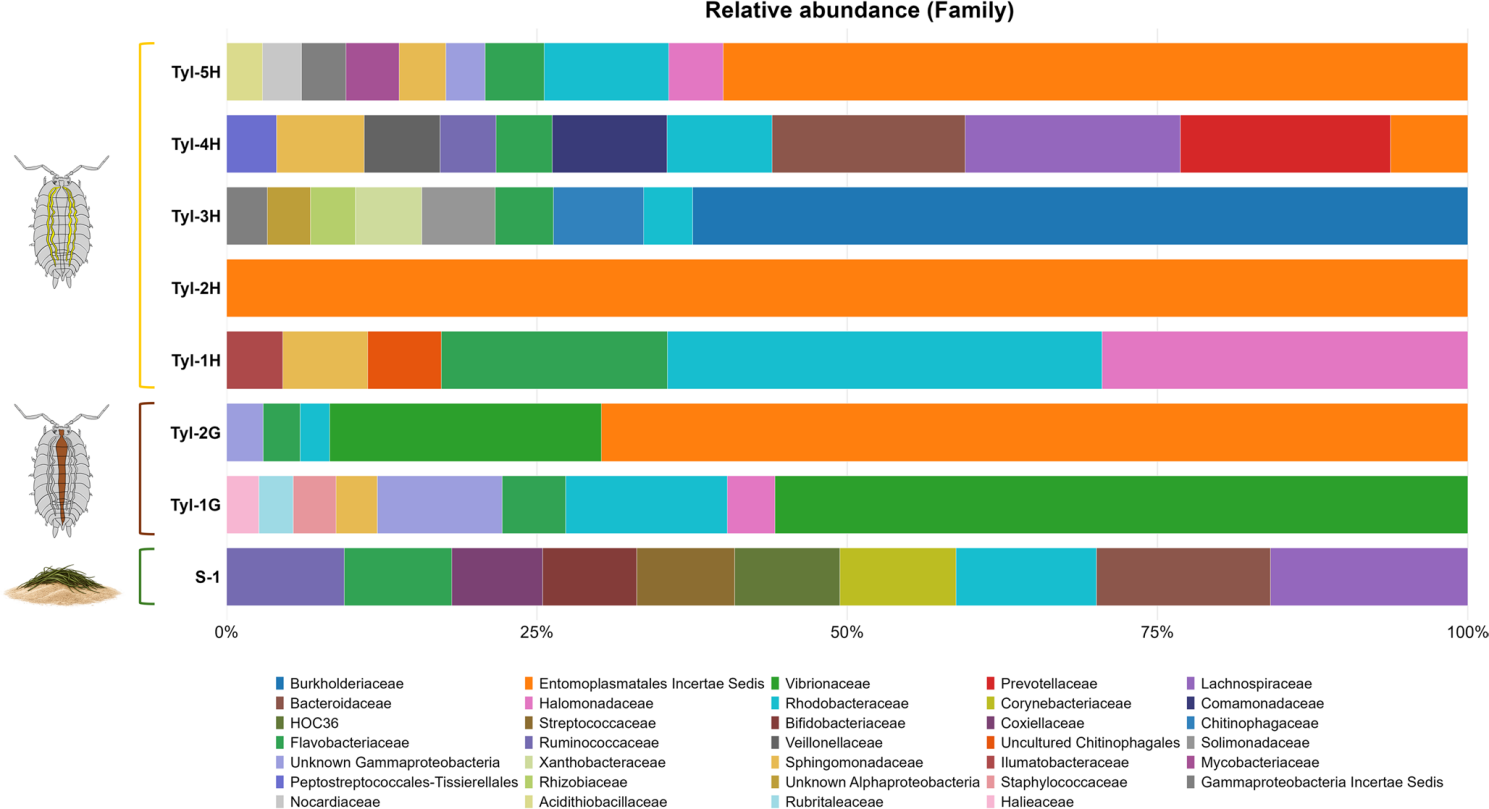
Taxonomic composition based on relative abundances of ASVs at the family level from five hepatopancreas (Tyl-1H–5H) and two guts (Tyl-1G-2G) of *Tylos ponticus* and a sample of sand (S-1). All ASVs with a relative count below 2% in each sample were removed. Illustrations on the left highlight the sample types examined (yellow=hepatopancreas; brown=gut; green=*Posidonia* banquette sand).

A total diversity of 100 ASVs at the family level (Fig. 4; Suppl. Table S3) and 157 ASVs at the genus level (Fig. 5; Suppl. Table S4) were observed with a relative abundance ≥ 2% in each sample. Proteobacteria families were overwhelmingly predominant with Vibrionaceae (genus *Vibrio*), Halomonadaceae (genus *Cobetia*), and Rhodobacteraceae (unknown genus) being the most abundant in almost all the samples.

#### Environmental Bacteria

An extensive metabarcoding analysis of the microbial communities associated with the supralittoral *Posidonia* banquette lies beyond the scope of the present study. The inclusion of the sand sample was not intended to support a formal host–environment comparison, but rather to identify and exclude taxa also present in the surrounding substrate, thereby allowing us to focus the discussion on microbial components more likely associated with *Tylos* (Figs. 4 and 5; Supplementary Tables S3-S4). At the genus level, *Candidatus* Hepatoplasma was present in all analysed samples (except Tyl-3H) and was also relatively abundant in the environmental sample (S-1). Similarly, *Vibrio* (family Vibrionaceae), *Sphingomonas* (Sphingomonadaceae), and an unidentified *Rhodobacteraceae* were detected across multiple samples. In contrast, certain bacterial taxa were found exclusively in the sand sample. These included *Coxiella* (family Coxiellaceae), *Vermiphilus* (Vermiphilaceae), *Woeseia* (Woesiaceae), and *Candidatus* Peribacteria *(*Bacteria; *Candidatus* Peregrinibacteriota), which together accounted for more than 11% of the bacterial variability detected in the environmental sample. Particularly worthy of note is the presence of *Bdellovibrio* (Bdellovibrionaceae) known for their parasitic behaviour [46]. 

#### Hepatopancreas and Hindgut Bacteria

**Fig. 6:**
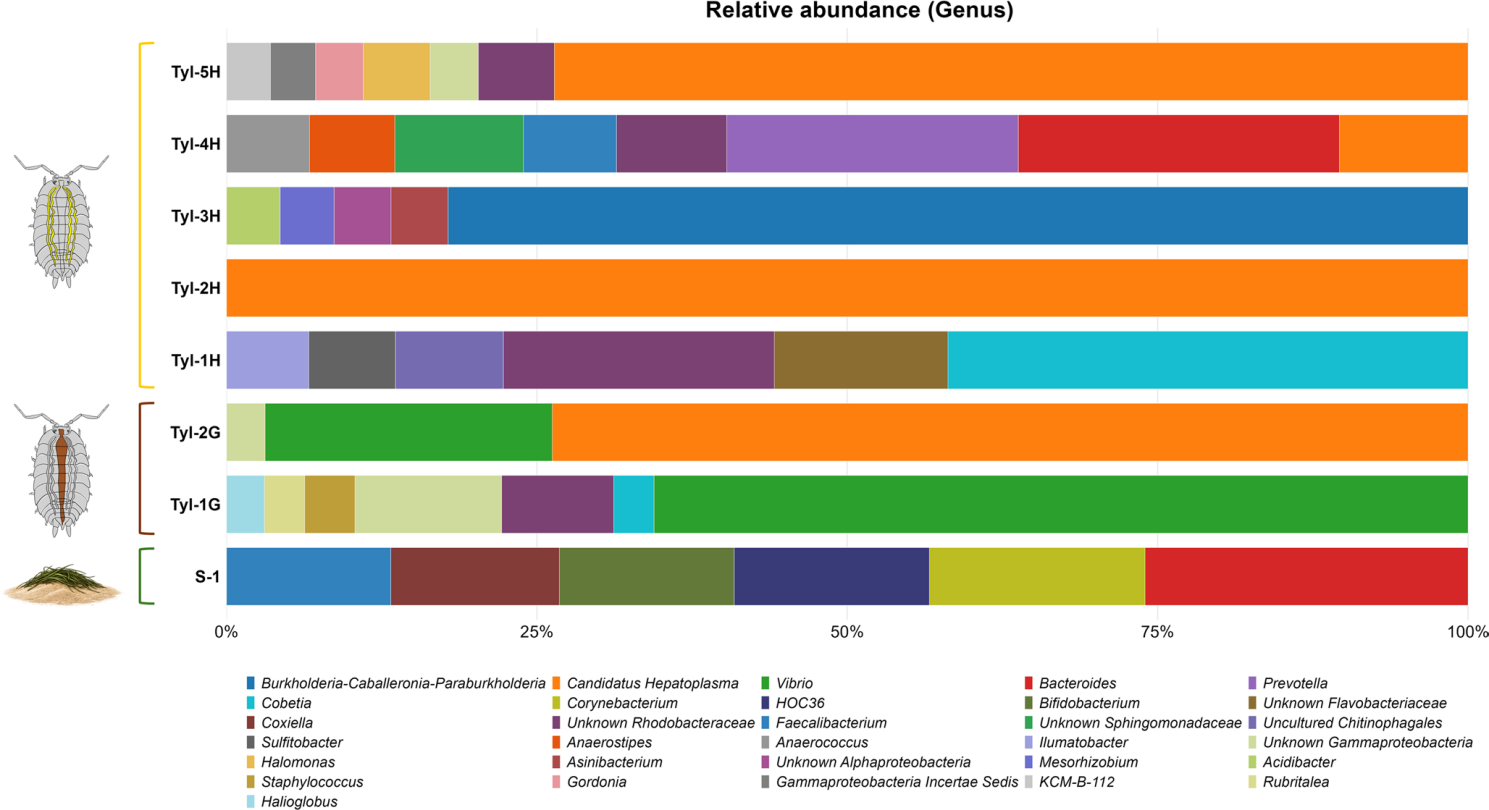
Taxonomic composition based on relative abundances of ASVs at the genus level from five hepatopancreas (Tyl-1–5H) and two guts (Tyl-1-2G) of *Tylos* *ponticu*s and a sample of sand (S-1). All ASVs with a relative count below 2% in each sample were removed. Illustrations on the left highlight the sample types examined (yellow=hepatopancreas; brown=gut; green=*Posidonia* banquette sand)

Within the hepatopancreas and gut of the crustacean species, we focused on ASVs not detected in the S-1 sand sample, in order to identify microbial taxa potentially associated with the host. Given the limited number of gut and environmental samples, these observations should be considered exploratory and do not allow for definitive conclusions on host specificity or symbiotic interactions. We identified 214 different genera exclusively present in at least one hepatopancreas sample (Supplementary Table S2). Among the most abundant were *Oceanospirillum* (Oceanospirillaceae), *Nitriliruptor* (Nitriliruptoraceae), *Oceanococcus* (Solimonadaceae), *Acquabacterium* (Comamondaceae), *Cellulophaga* and *Zobellia* (Flavobacteriaceae), and *Halarcobacter* (Arcobacteraceae). Also noteworthy for their functional roles were *Photobacterium* (Vibrionaceae), *Marinobacter* (Alteromonadaceae), *Phaeobacter* (Rhodobacteraceae), and *Leucothrix* (Leucotrichaceae). The 50 most abundant genera shared by both *T. ponticus* internal organs (Table S2), along with their relative count frequencies, are presented in the heatmap in Fig. 6. Among them, *Cobetia* (Halomonadaceae) was the most abundant, followed by a Burkholderiaceae group composed of *Burkholderia*, *Caballeronia*, and *Paraburkholderia*. Several other bacterial taxa were common to both the gut and hepatopancreas, including *Psychrobacter* (Moraxellaceae), *Roseovarius* (Rhodobacteraceae), and *Halomonas* (Halomonadaceae).

#### Alpha and Beta Diversity Analysis

Based on 16S rRNA gene sequencing, alpha diversity indices (Chao1, Shannon, and Simpson) showed comparable values across most microbiota samples, with the exception of Tyl-2H and S-1, which exhibited the lowest and highest diversity, respectively. The indices were calculated based on family- and genus-level abundances across all samples. The Chao1, Shannon, and Simpson values for the remaining gut and hepatopancreas samples confirmed the high similarity in ASV composition and relative abundance (Table 1), with Tyl-4H displaying the highest overall richness among hepatopancreas samples.

**Table 1:** The alpha diversity evaluated using Simpson, Shannon and Chao1 indices for microbiota of and the hepatopancreas of *Tylos ponticus* (Tyl-1–5H), for its gut (Tyl-1G–2G) and for the microbial community of sand beach (S-1). Average values based on family (right) and genus (left) composition and relative abundance are reported

	Simpson		Shannon		Chao1	
	Gen	Fam	Gen	Fam	Gen	Fam
S-1	0,99	0,98	6,80	6,35	141	109
Tyl-1H	0,95	0,90	5,65	4,41	130	74
Tyl-1G	0,78	0,77	3,78	3,41	64	46
Tyl-2H	0,10	0,10	0,52	0,48	37	27
Tyl-2G	0,62	0,61	2,44	2,33	41	30
Tyl-3H	0,83	0,83	4,55	4,25	95	75
Tyl-4H	0,98	0,96	6,02	5,31	93	64
Tyl-5H	0,81	0,81	4,45	4,07	110	81

**Fig. 7:**
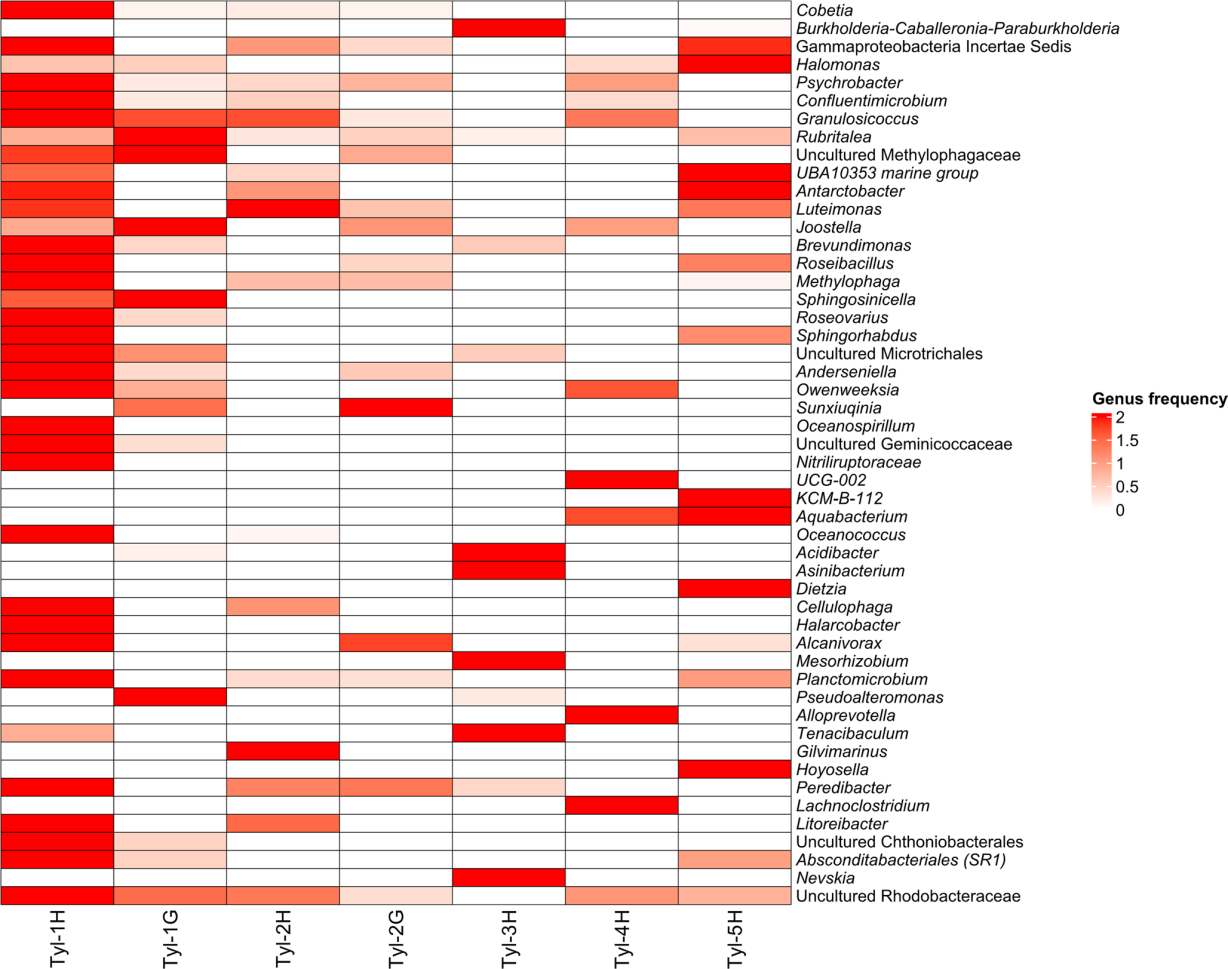
Frequency heatmap depicting the differences in the relative abundance of the 50 most frequently common bacterial genera among hepatopancreas and guts samples. The colour intensity in each cell of the matrix reflects the frequency of the feature in the corresponding sample. White=not present.

The analyses of beta diversity showed that the microbiota from hepatopancreas and the gut of the crustacean species and that of the supralittoral sand did not form distinct groups in terms of the taxonomic composition. The PCoA based on Bray-Curtis distances carried out on the ASV datasets at the family and genus levels from the microbiota (Fig. 7) shows a lack of distinct clustering among most of the samples, emphasising the heterogeneity of the microbial communities in the dataset. The percentages of variance for the first three principal components are the following: PCoA family: PC1=28.8% PC2=20.0%, PC3=18.5%. PCoA genus: PC1=26.3%, PC2=20.1%, PC3=18.4%.

### Whole Genome Sequencing of* Vreelandella Venusta* Strain H3

We obtained bacterial cultures from the hepatopancreas microbiome of *Tylos ponticus* with the potential of ligninolytic activity, which were selected and taxonomically classified. In particular, we focused on the bacterial strains potentially related to the production of lignocellulose-degrading CAZymes, which might be uniquely adapted within the hepatopancreatic lumen. In this study, we present the draft genome sequence of *Vreelandella* sp. strain H3 (GC content 52.5%). The genus *Vreelandella*, family Halomonadaceae, order Oceanospirillales, class Gammaproteobacteria was recently delineated from the genus *Halomonas* [47]. Currently, *Vreelandella* comprises several species that were originally classified under *Halomonas*, including strain H3.

Average Nucleotide Identity based on BLAST (ANIb) analysis revealed that strain H3 shares 98.40% identity with *Vreelandella venusta* DSM 4743^T (Assembly Accession: GCA_016107635.1; genome length: 4,307,261 nt; GC content: 52.66%) and 98.41% with *Vreelandella venusta* NBRC 102,221 (Assembly Accession: GCA_007989605.1; genome length: 4,273,738 nt; GC content: 52.62%). According to JSpecies analysis, these ANIb values strongly support the classification of strain H3 within the species V*reelandella venusta*.

The sequencing of *Vreelandella venusta* strain H3 generated 2,042,915 paired-end reads. The genome assembly resulted in a set of scaffolds totaling 4,155,009 base pairs. The assembled genome has been deposited in GenBank under BioProject accession PRJNA860326, BioSample SAMN29861931, and Sequence Read Archive (SRA) SRR20391950. Genome annotation predicted a total of 4,217 coding sequences (CDSs), 62 tRNA genes, and three complete rRNA operons, including two 5S rRNA genes and one operon comprising 5S, 16S, and 23S rRNA genes.

**Fig. 8:**
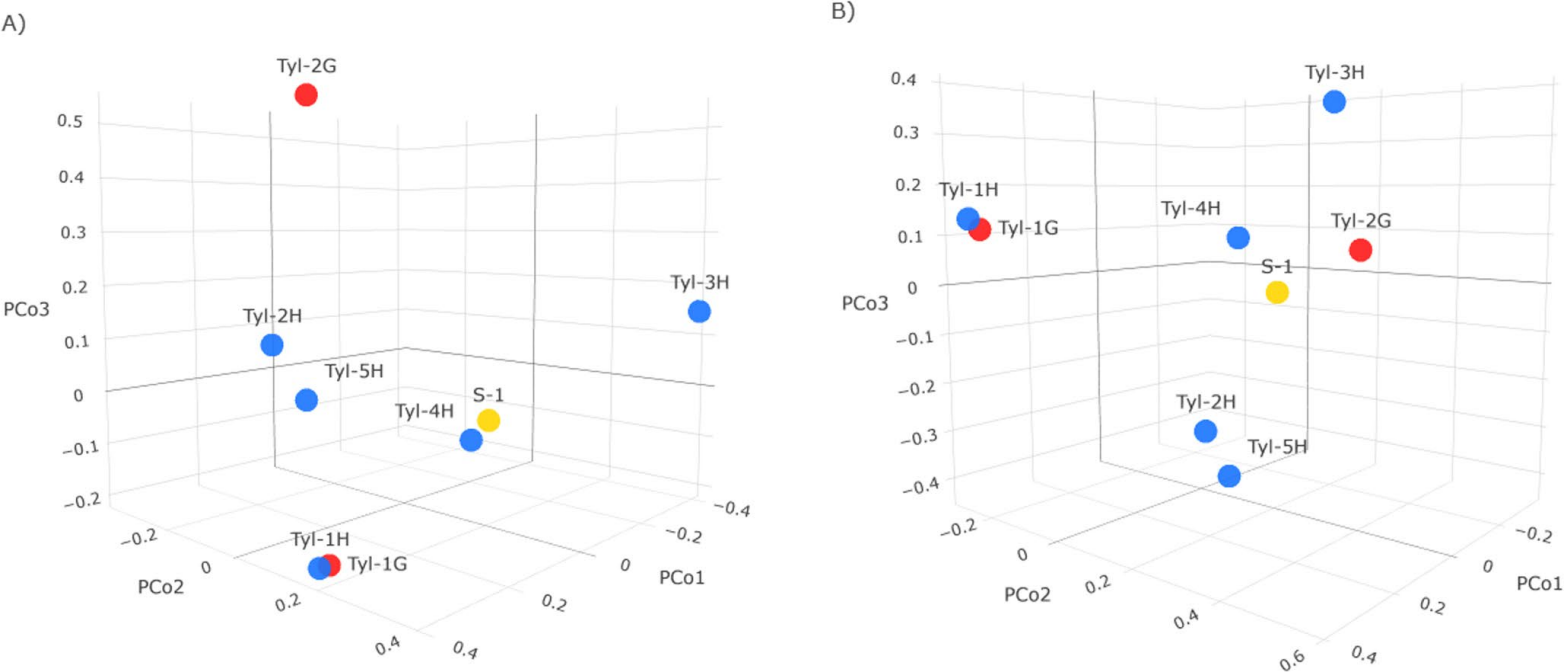
Beta diversity of the microbiota found in the *Tylos ponticus* hepatopancreas, in their guts and in the supralittoral sand. Bray-Curtis distances were calculated based on the family (A) and genus (B) composition and relative abundance and then visualised through Principal Coordinates Analysis. Yellow=sand, Blue=hepatopancreas, Red=gut

 A comparative genomic analysis was performed on the strain H3 found in the hepatopancreas of *T. ponticus* (Fig. 8) alongside related genome sequences from species within the genera *Halomonas* and *Vreelandella* available in GenBank. The analysis specifically focused on identifying genes potentially involved in lignocellulose degradation, including pathways targeting cellulose, hemicellulose, and lignin. The *de novo* assembly and annotation of the genome of *V. venusta* strain H3 revealed the presence of several genes associated with lignocellulose degradation such as genes encoding Auxiliary Activities (AAs), Carbohydrate Esterases (CEs), Glycoside Hydrolases (GHs), and Glycosyl Transferases (GTs; Table S5). In particular, hydrolytic enzymes targeting plant cell walls were identified, such as β-glucosidases (family GH3) specific for cellulose. Additionally, an α-glucosidase (family GH31) acting on starch was detected.

Other CAZymes identified and classified in the CAZy database included two laccases (family AA1), a lignin peroxidase (family AA2), two cellobiose dehydrogenases (family AA3), two quinone oxidoreductases (family AA6), another glycoside hydrolase from family GH31, and three hemicellulases from family CE1. Notably, lignin-modifying enzymes and oxidative cellulases from the AA families, including members of the AA3 family, were present in the genome of *V. venusta* strain H3, supporting its potential role in lignocellulose degradation (Table S5).

 AntiSMASH analysis of the *V. venusta* H3 genome identified seven biosynthetic gene clusters (BGCs), including polyketide synthases (PKSs) and nonribosomal peptide synthetases (NRPSs). Among these, the ectABC cluster encoding ectoine biosynthetic enzymes was predicted. Comparison with the MIBiG database showed that Region 1 (Table S6) is most similar to the ectoine BGC from *M. kenyense* (BGC0000855). The ectABC operon comprises *ectC* (ectoine synthase), *ectB* (diaminobutyrate—2-oxoglutarate transaminase, EC 2.6.1.76), and *ectA* (diaminobutyrate acetyltransferase), with an upstream open reading frame encoding a transcriptional repressor. The synteny and homology of this cluster with those in *Halomonas hydrothermalis* (NZ_CP023656.1.region003), *Halomonas meridiana* (NZ_CP024621.1.region001), and *Halomonas piezotolerans* (NZ_CP048602.1.region001) are detailed in Supplementary Figure S2.

### Shotgun Reads

Shotgun metagenomic libraries from the hepatopancreas of *Tylos ponticus* (samples Tyl-2H and Tyl-4H) were validated and quantified with Bioanalyzer 2100 and with a Qubit 2.0 Fluorometer. The total number of *T. ponticu*s Illumina reads obtained for each library ranged from 39,426,338 (Tyl-2H; Supplementary Table S7) to 47,463,340 (Tyl-4H; Supplementary Table S7). The results of the metagenomic profiling from the hepatopancreas of *T. ponticus*, specimens Tyl-2H and Tyl-4H, visualized as a pie chart using Krona [37] are reported in Supplementary Figure S3.

Although *Candidatus* Hepatoplasma reads were a minor fraction of the Tyl-4H dataset, we recovered its metagenome‑assembled genome (MAG) by de novo assembly (MEGAHIT; SPAdes v4.2.0) with a minimum contig length of 1 kb, polishing (Pilon), and binning (MetaBAT2).

Quality assessment by Quast v5.3.0 revealed that the *Ca.* Hepatoplasma MAG had completeness < 90%, contamination > 5% and uneven GC content, disqualifying it as a high‑quality MAG and precluding robust ANI comparisons (JSpecies [28]). Instead, 16S rDNA and rpoD‑based BLAST analyses (NCBI BLAST) indicated closest identity to *Candidatus* Hepatoplasma vulgare Av‑JP [9]. Since a complete assembly of the targeted *Candidatus* Hepatoplasma genome was not obtained, an average nucleotide identity-BLAST (Genome ANIb [%]) between *Candidatus* Hepatoplasma cf. vulgare Tp and other uncultured *Candidatus* Hepatoplasma species from isopods recently available at GenBank, NCBI [9] was not performed.

However, BLAST analysis (available at NCBI website, https://blast.ncbi.nlm.nih.gov/Blast.cgi accessed on May 2024) based on the 16S rDNA sequence and other taxonomic genomic marker genes such as *rpoD* gene extracted from our *Candidatus* Hepatoplasma genome indicated that this taxon is closely related to *Candidatu*s Hepatoplasma vulgare Av-JP [9]. Nevertheless, the draft assembly of *Candidatus* Hepatoplasma of *T. ponticu*s was compared with the shotgun sequencing data of *Candidatus* Hepatoplasmataceae members that include *Candidatus* Tyloplasma litorale Fukuoka 2020 [GenBank Accession Number (GAN): AP027078.1] from the semiterrestrial isopod *Tylos granuliferus*, *Candidatus* Hepatoplasma vulgare *Av-JP* (GAN: AP027131.1) from the terrestrial isopod *Armadillidium vulgare*, Candidatus Hepatoplasma scabrum Ps-JP (GANr: AP027133.1) from the terrestrial isopod *Porcellio scaber*, *Candidatus* Hepatoplasma crinochetorum Tokyo 2021 (GAN: AP027132) identified in the shotgun sequencing data of *A. vulgare*, and *Candidatus Hepatoplasma crinochetorum Av* from *A. vulgare* (GAN: CP006932). *Mycoplasmataceae* bacterium isolate DT_51 (GAN: JABSQD000000000.1) and *Mycoplasmataceae* bacterium isolate GLR43, (GAN: WTBJ00000000.1) of the family *Mycoplasmataceae* (Bacteria; Bacillati; Mycoplasmatota; Mollicutes) were used as outgroup. The genome sequence of *Candidatus* Hepatplasma of *T. ponticu*s appears to be reduced in line with other *Candidatus* Hepatoplasma genomes [9, 39, 48].

**Fig. 9:**
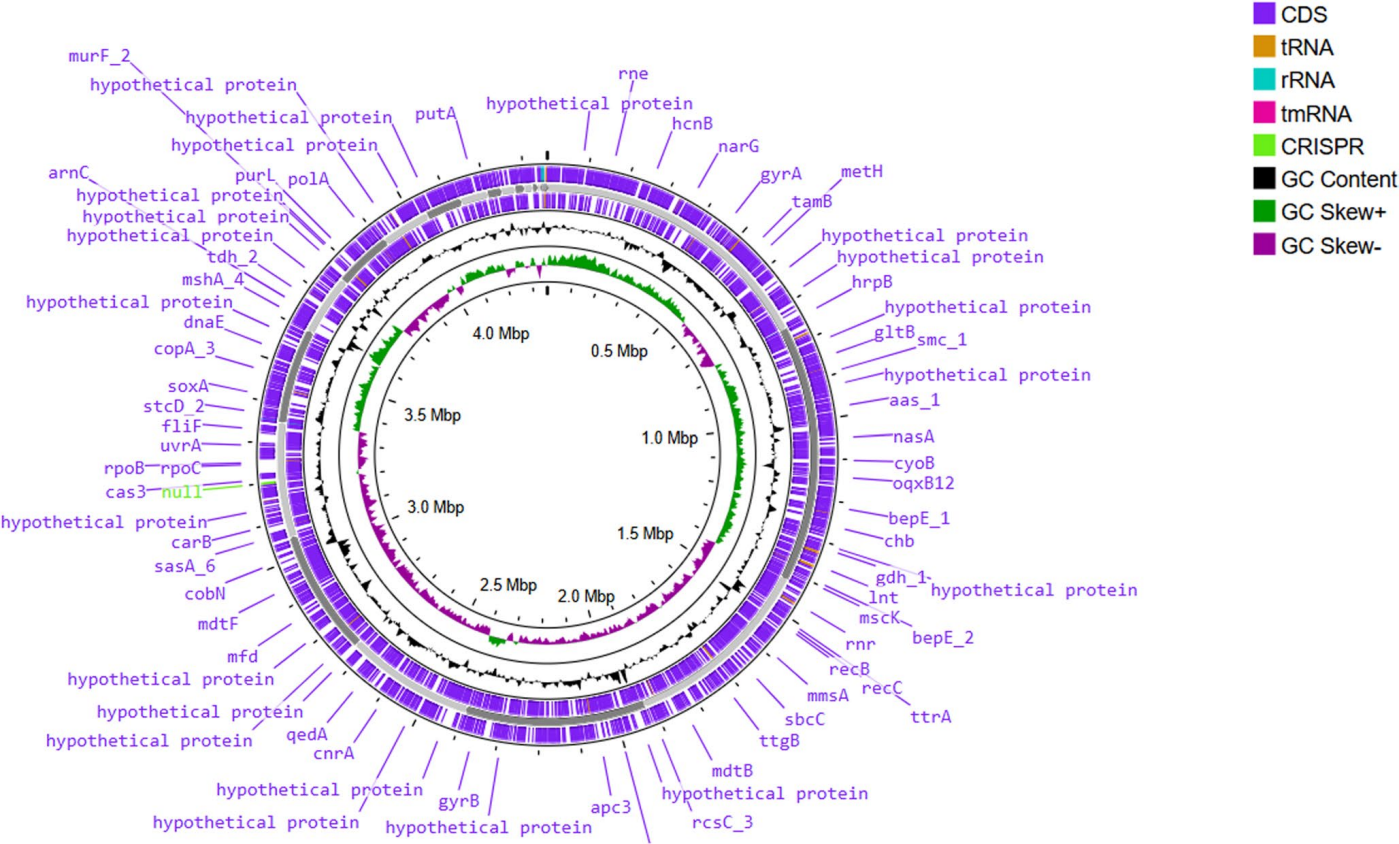
Circle chromosome genome of *Vreelandella venusta* strain H3 formed by Proksee. GC skew (innermost circle), GC content (second circle), and original features (i.e. CDS, tRNA, rRNA) are shown. Original features, such as CDS outside of the backbone (gray) are from positive strand (+) of DNA, and the original features shown inside of backbone are from negative strand (-) of DNA

 The phylogenomic tree based on the identification of highly conserved single-copy genes, describes the evolutionary relationships between species of the *Candidatus* Hepatoplasmataceae family. It is noteworthy that according to our phylogenomic analysis (Fig. 9), *Candidatus* Hepatoplasma of *T. ponticu*s is closely related to *Candidatu*s Hepatoplasma vulgare Av-JP [9]. *Candidatus* Hepatoplasma scabrum Ps‑JP belong to the same clade of *Candidatus* Hepatoplasma crinochetorum Tokyo 2021, whereas *Candidatus* Tyloplasma litorale Fukuoka 2020 grouped with Mycoplasmataceae taxa, forming a distinct monophyletic lineage. However, given the incomplete nature of the assembly, this result should be regarded as preliminary. Functional annotation of the draft genome suggested a reduced metabolic repertoire, including a limited set of carbohydrate-active enzymes (CAZymes), identified through DIAMOND searches against the CAZy database (Table S8). However, given the incomplete nature of the assembly, the absence or low abundance of specific functional categories should be interpreted with caution.

Interestingly, the draft genome *Candidatus* Hepatoplasma of *T. ponticu*s encodes three CRISPR‑associated genes—cas1, cas2 and cas9—consistent with a type II CRISPR/Cas system [39]. Notably, cas1 and cas2 are located separately from cas9 (data not shown), and neither csn2 nor cas4 homologs were detected. In addition, antiSMASH v8.0 analysis revealed no secondary‑metabolite biosynthetic gene clusters in this draft assembly.

## Discussion

### *Tylosponticus* Complex

The phylogenetic tree based on mitochondrial and nuclear gene sequences showed that the *Tylos* specimens of this study belonged to a single genetic clade of *T. ponticus* (Fig. 3) clearly genetically distinct from *T. europaeus* and *T. capensis*. However, it is unclear if the genetic differentiations observable among *T. ponticus* specimens is due to intraspecific coalescence or interspecific divergence, as often occurs in othergroups of invertebrates (e.g. [49]). As previously suggested by [5], *T. ponticus* likely represents a complex of two cryptic species: one including Portuguese and Greek samples, and the other represented by the Libyan specimens, which in our study cluster within the same clade as the Italian samples. Further investigation would be valuable, both from genetic and morphological perspectives, for a better understanding of the evolution within the *T. ponticus* complex.

### Histological Investigation of the Hepatopancreas

The histological observations were included to provide morphological context for the hepatopancreatic environment where the symbionts reside, and to confirm the presence of typical isopod hepatopancreatic cell types, which are known to be involved in digestion and potentially in host–microbe interactions. B and S cells were clearly distinguished in the hepatopancreas of *Tylos ponticus* (Fig. 4), as previously reported for other isopods [6, 50]. The ultrastructural features observed in the B cells, including the microvillar apical surface and the presence of cytoplasmic vacuoles, are consistent with those previously described in other isopods such as *Armadillo officinalis* Duméril, 1816 [51]. The S cells cells were more compact with a densely granular cytoplasm and likely are responsible for secretion and the production of digestive enzymes as previously described in isopods [6]. Our results allowed to reveal functionally active cells in the hepatopancreas of *T. ponticus*, some of which (B cells) exhibited a vacuolated cytoplasm, a prominent apical brush border and cell secretion activity, protruding apically into the hepatopancreatic lumen. Additionally, vegetable materials were clearly distinguished inside the gut tracts of *T. ponticus* (Fig. 4), revealing that in this species, like in other oniscidean isopods, the finest plant material is filtered through specialized systems and then the fluids are directed to the hepatopancreatic tubules.

### Structure of Bacterial Communities by 16S rRNA Metabarcoding

Our metabarcoding analysis of the bacterial communities associated with the hepatopancreas and hindgut of *Tylos ponticus* revealed a rich and diverse microbiota dominated by Proteobacteria, particularly members of the families Vibrionaceae, Halomonadaceae, and Rhodobacteraceae. Members of the family Hepatoplasmataceae were also detected, appearing in the barplot analysis under the taxonomic assignment *Entomoplasmatales* incertae sedis (Figs. 4 and 5; Tables S3 and S4). These results are consistent with patterns previously observed in other isopod species (e.g. [52]), as well as in other marine invertebrates (e.g. [24, 53]).

While some bacterial taxa were shared between the environmental sample and the *T. ponticus* microbiota, several genera were detected exclusively in the environmental sample (Fig. 5, Table S4). These included genera *Coxiella* that comprises species with zoonotic potential [54], as well as *Vermiphilus*, reported as an amoeba-associated parasite [55], *Woeseia*, a widespread constituent of marine microbial communities [56], and *Candidatus* Peribacteria, originally described from an alluvial aquifer system [57]. Notably, the environmental sample also contained *Bdellovibrio* (Bdellovibrionaceae), a predatory bacterium that attacks other Gram-negative bacteria [46]. Given that only a single environmental sample was analysed, these observations should be interpreted conservatively. However, the detected environmental are consistent with taxa reported in similar environments. Indeed, some of the genera detected in the sand (e.g., *Coxiella*, *Vermiphilus*, and *Woeseia*) have also been reported in gut-associated datasets of other isopods, including *Porcellionides pruinosus* [58], although their occurrence across habitats may reflect broad environmental distributions rather than obligate host association. In particular *Coxiella* was abundant in the gut bacterial communities of the isopod *Porcellinoides pruinosus* (Brandt, 1833) [58], and *Coxiella*-like endosymbionts have evolved into nutritional symbionts in ticks [59]. More generally, supralittoral sands provide heterogeneous microhabitats that sustain diverse microbial consortia supported by organic inputs; in these settings, taxa such as *Vermiphilus* (including members affiliated with *Candidatus* Babelota) have been linked to protist-associated assemblages [60], and *Woeseia* has been reported as a recurrent marine microbial partner. Predatory lineages such as *Bdellovibrio* are also common components of microbial food webs and may play a role in structuring supralittoral communities [61, 62].

Our data show an overlap of bacterial genera between the gut and hepatopancreas of *T. ponticus*, suggesting the presence of potentially shared microbial taxa. However, given the limited number of gut samples, this pattern does not allow us to robustly define a core gut microbiota. The heatmap (Fig. 6) highlights genera such as *Cobetia* (Halomonadaceae) and a Burkholderiaceae group (formed by *Burkholderia*, *Caballeronia* and *Paraburkholderia*) of Betaproteobacteria as particularly abundant in both hepatopancreas and gut of *T. ponticus*. *Cobetia* is typically found in littoral environments, where it plays an important role in algal degradation, while genera belonging to the family Burkholderiaceae are ubiquitous across various habitats, including marine environments [24, 63, 64] and *Paraburkholderia* symbionts were isolated from the amoeba *Dictyostelium* [65]. Other commonly shared bacterial genera included *Psychrobacter* (Moraxellaceae), *Roseovarius* (Rhodobacteraceae), and *Halomonas* (Halomonadaceae), all of which have already been reported in marine invertebrates [4, 66].

However, the hepatopancreas hosted 214 genera unique to one or more samples, such as several that play key roles in marine ecosystem functioning. Among them, *Oceanospirillum* and *Marinobacter* are known for their hydrocarbon-degrading capabilities in coastal and deep-sea environments [67, 68]. *Nitriliruptor* species, isolated from hydrothermal vents, contribute to nitrogen cycling through nitrile metabolism [69]. *Cellulophaga* and *Zobellia* are efficient degraders of algal polysaccharides, supporting carbon turnover in marine habitats [70, 71]. *Photobacterium* includes both bioluminescent symbionts and marine pathogens [72], while *Phaeobacter* produces antimicrobial compounds that may shape host-associated microbiomes [73]. *Leucothrix* and *Halarcobacter* are involved in sulfur cycling and are often found on marine surfaces [74, 75]. Finally, the ecological role of *Oceanococcus* remains poorly understood but it is frequently detected in saline marine environments [76], while *Acquabacterium* is also common in drinking water [77].

A key finding of our study is the widespread occurrence of *Candidatus* Hepatoplasma in nearly all analysed hepatopancreas and hindgut samples of *T. ponticus*, as well as in the surrounding sediment, though at varying abundances. Its absence in a single hepatopancreas sample (Tyl-3H) may reflect biological or sampling variation. *Candidatus* Hepatoplasma has been previously identified as a dominant symbiont in the hepatopancreas of both terrestrial and aquatic crustaceans [78, 79]. Overall, its consistent detection in *T. ponticus* suggests a stable and potentially symbiont association and could be primarily dictated by *Tylos* constraints, reflecting *Tylos*-*Candidatus* Hepatoplasma trade-offs (see below). However, *Candidatus* Hepatoplasma was not confined to *T. ponticus* niches, with its presence in the sand sample, although at lower abundance. Investigating faeces bacterial communities of *T. ponticus* in the environment using high-throughput sequencing could help provide deeper insights into the life cycle and evolution of *Candidatus* Hepatoplasma in isopods.

Based on 16S rRNA gene sequencing, alpha diversity indices (Table 1) showed comparable values across most microbiota samples, with the exception of Tyl-2 H (hepatopancreas), which exhibited the lowest diversity, and S-1 (sand), which displayed the highest value. The diversity indices of the gut microbiota from the two specimens confirmed a high similarity of their ASVs in terms of both composition and relative abundance. In contrast, greater variability was observed among hepatopancreas samples, with Tyl-4H showing the highest taxonomic richness. Beta diversity analyses revealed that the microbial communities associated with the hepatopancreas and gut tissues of *T. ponticus*, as well as those from the surrounding sediment, did not form distinct clusters based on taxonomic composition. Indeed, the PCoA plots (Fig. 7) revealed a diffuse distribution of samples, indicating a high degree of heterogeneity across the dataset. The lack of distinct clustering suggests that the hepatopancreas and gut microbiota of *T. ponticus* are not strongly compartmentalized, which may be influenced by specific conditions such as the vegetable fragments ingested. In this context, future investigations should also consider the fungal component (mycobiome) of these microbial communities, which is increasingly recognized as an integral part of the hepatopancreas and gut microbiota of terrestrial isopods [80].

### *Vreelandella Venusta* Strain H3

The genus *Vreelandella*, formerly classified within *Halomonas* (family Halomonadaceae [81, 82]), includes halotolerant strains capable of degrading polysaccharides and environmental pollutants, as well as producing polyhydroxyalkanoates [83, 84]. Whole genome sequencing of *V. venusta* strain H3, isolated from the hepatopancreas of *T. ponticus*, suggests that members of Halomonadaceae contribute to lignocellulose degradation through carbohydrate-active enzymes (CAZymes) in supralittoral oniscidean isopods. Our results reveal the potential role of *V. venusta* H3 in lignin degradation, supported by the presence of bacterial genes encoding lignin-modifying enzymes from the AA families within its genome (Table S5). Previous studies have identified lignin-degrading genes, including catalases and peroxidases, in *Halomonas* spp [85, 86]., and *Vreelandella* (formerly *Halomonas*) was found among the most abundant genera in the *Armadillidium vulgare* microbiome [8]. These findings suggest that the *T. ponticus* holobiont, combining endogenous CAZymes and symbiotic bacteria like *V. venusta* H3, may effectively degrade lignin into cellulose and hemicellulose [8, 20].

AntiSMASH analysis of the *V. venusta* H3 genome identified seven BGCs, including PKSs and NRPS. Among these, the ectABC cluster encoding ectoine biosynthetic enzymes was predicted (Supplementary Table S6; Figure S2). These results suggest *V. venusta* H3’s potential to produce ectoine, a compatible solute synthesized by halophilic *Halomonas* and *Vreelandella* strains to mitigate high salinity stress [84]. However, experimental validation of ectoine production in H3 is needed [87, 88]. Notably, industrial scale ectoine production has been achieved using *Halomonas elongata* [89] and through heterologous expression of the *ectABC* cluster from *Halomonas venusta* in *Escherichia coli* [90].

Understanding halophilic symbionts in the *Tylos*-microbe system may have broad biotechnological implications, particularly for sustainable ectoine production from marine biomass as well as for the potential role of the oniscidean isopod gut microbiota in plastic degradation in marine ecosystems.

### *Candidatus *Hepatoplasma from *Tylos ponticus*

Genome shotgun sequencing confirmed for the first time the presence of *Candidatus* Hepatoplasma in the hepatopancreas of *T. ponticus*. *Candidatus* Hepatoplasma, as uncultured symbiont, is typically found on the brush borders (that consist of microvilli extending into the lumen) of the hepatopancreatic B cells in semiterrestrial and terrestrial isopods [51]. *Candidatus* Hepatoplasma crinochetorum, for example, has been identified in several isopod species, including *T. europaeus* [39].

**Fig. 10:**
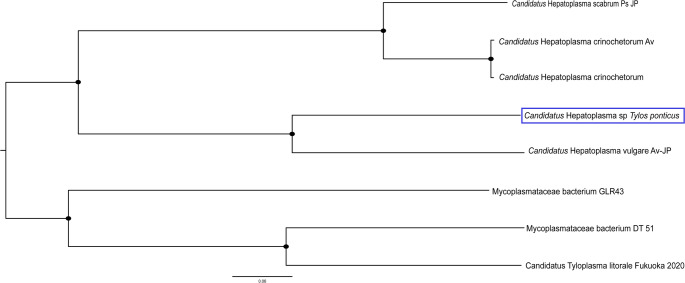
A phylogenomic tree (tree method: Maximum likelihood Evolution, Model: JTT+CAT) that illustrates the evolutionary relationships among *Candidatus* Hepatoplasma cf. vulgare Tp and MAGs of *Candidatus *Hepatoplasmataceae members from isopods. Black circles at nodes indicate maximum support (PP=1). In blue, sample sequences generated in this study

Our phylogenomic analysis (Fig. 10) shows that *Candidatus* Hepatoplasma of *T. ponticus* belongs to the family *Candidatus* Hepatoplasmataceae and is associated with isopods. Within this family, it appears as the closest relative of *Ca.* Hepatoplasma vulgare Av-JP, previously identified in *Armadillus vulgare*. *Ca.* Hepatoplasma scabrum Ps-JP grouped with *Ca.* Hepatoplasma crinochetorum Tokyo 2021, while *Ca.* Tyloplasma litorale Fukuoka 2020 formed a separate clade together with members of the family *Mycoplasmataceae*. *Ca.* Tyloplasma litorale, isolated from the semiterrestrial isopod *Tylos granuliferus*, represents a lineage distinct from *Ca.* Hepatoplasma species. Its placement in an independent clade supports an evolutionary divergence within the *Candidatus* Hepatoplasmataceae, suggesting lineage diversification in the isopod genus *Tylos*. Our results are consistent with previously published data based on metagenome-assembled genomes of *Candidatus* Hepatoplasmataceae [9]. As our analysis showed a close relationship between *Candidatus* Hepatoplasma *from T. ponticus* and *Ca.* Hepatoplasma vulgare Av-JP [9], but definitive identification could not be confirmed, we adopted the provisional designation cf. to indicate the need for further taxonomic validation. Based on the data obtained from *T. ponticus*, we propose the provisional taxonomic assignment *Candidatus* Hepatoplasma cf. vulgare Tp, within the genus *Candidatus* Hepatoplasma, family *Candidatus* Hepatoplasmataceae, order Mycoplasmatales, class Mollicutes, kingdom Mycoplasmatota, and domain Bacillati, in the bacterial lineage.

Bacteria engage in a variety of symbiotic relationships that often provide significant adaptive advantages to their hosts, in line with the holobiont concept [8]. However, investigating uncultured symbionts such as *Candidatus* Hepatoplasma remains challenging [20]. In this study, the functional role of *Candidatus* Hepatoplasma species detected in the hepatopancreas of *T. ponticus* remains unclear, as is the case for other uncharacterized *Candidatus* Hepatoplasma species associated with isopods. Our metagenomic analysis revealed only a limited number of putative CAZymes suggesting that this symbiont may not be involved in carbohydrate degradation. Nonetheless, it may play other roles in host–microbe interactions. One possible function could involve attachment to the microvilli-covered surface of B cells forming the brush border. Our histological analysis revealed that the epithelial B cells of *T. ponticus* are consistently covered by a dense brush border likely composed of tightly packed microvilli (Fig. 4). Indeed, previous ultrastructural studies have demonstrated that *Candidatus* Hepatoplasma species can adhere to the microvilli of B cells [51].

The draft genome of *Ca. Hepatoplasma* cf. vulgare Tp includes several hypothetical proteins with unknown function. Some of these may be targeted to host cellular compartments such as the endoplasmic reticulum, where they could undergo further processing, secretion, or act at the host–microbe interface. Nonetheless, the specific mechanisms underlying the adhesion of *Ca.* Hepatoplasma spp. and other symbionts to B-cell microvilli, as well as the nature of their interactions with host tissues, remain unresolved and lie beyond the scope of the present work. Additionally, while CRISPR-Cas systems may offer adaptive immunity, it remains unclear whether this system is still functional or has been recently inactivated in *Candidatus* Hepatoplasma spp [39].

Given these observations, further investigation is needed to clarify whether *Candidatus* Hepatoplasma sp. cf. vulgare Tp plays an active symbiotic role in the hepatopancreas of *T. ponticus*. A complete genome assembly of this taxon was not feasible within the scope of the present work due to resource limitations. However, we anticipate that the use of third-generation sequencing technologies and additional metagenomic analyses, incorporating reference genomes, will enable the production of a high-quality assembly. We also emphasize the importance of future comparative analyses, including synteny assessments and pairwise genome comparisons using average nucleotide identity based on BLAST, to better understand genome evolution within the *Candidatus* Hepatoplasmataceae family associated with oniscidean isopods.

## Supplementary Information

Below is the link to the electronic supplementary material.

Supplementary Material 1 (XLSX 26.8 KB)

Supplementary Material 2 (PNG97.4 KB)

Supplementary Material 3 (XLSX 9.41 KB)

Supplementary Material 4 (DOCX 13.9 KB)

Supplementary Material 5 (DOCX 14.3 KB)

Supplementary Material 6 (XLSX 15.0 KB)

Supplementary Material 7 (XLSX 13.5 KB)

Supplementary figure 8 (PNG 170 KB)

High Resolution Image (TIF 1.05 MB)

Supplementary Material 9 (XLSX 15.1 KB)

Supplementary Material 10 (DOCX 15.9 KB)

Supplementary figure 11 (PNG 1.36 MB)

High Resolution Image (TIF 3.15 MB)

Supplementary Material 12 (XLSX 45.0 KB)

## Data Availability

Genetic data: all the sequences are deposited to public repository NCBI [https://www.ncbi.nlm.nih.gov/](https:/www.ncbi.nlm.nih.gov).
