# Supplementary material for: Understanding Hepatopancreas-Associated Microbiota in the Supralittoral Tylos ponticus (Crustacea, Isopoda, Oniscidea): Insights from Next-Generation Sequencing Approaches
Source: Microb Ecol. 2026 May 23;89:125. doi: 10.1007/s00248-026-02785-4 (PMC13264560; doi:10.1007/s00248-026-02785-4)
Supplement: Supplementary file 1 [file 248_2026_2785_MOESM10_ESM.docx]

**Supplementary captions**

**Table S1**. Sequences list of *Tylos* spp. used in this work along with their Genbank accession numbers.

**Table S2**. The 50 most abundant genera shared by both *T. ponticus* hepatopancreas and guts identified through 16S metabarcoding analyses.

**Table S3**. The total number of ASVs identified at the family level in the five guts, two hepatopancreas of *T. ponticus* and in the sand samples identified through 16S metabarcoding analyses.

**Table S4**. The total number of ASVs identified at the genus level in the five guts, two hepatopancreas of *T. ponticus* and in the sand samples identified through 16S metabarcoding analyses.

**Table S5.** Carbohydrate-active enzymes (CAZymes), including Auxiliary Activities (AAs), Carbohydrate Esterases (CEs), Glycoside Hydrolases (GHs), and Glycosyl Transferases (GTs) in the genome of *Vreelandella venusta* strain H3 identified via dbCAN3 (Zhang et al., 2018; Zheng et al., 2023), integrating HMMER, DIAMOND, and Hotpep searches with stringent thresholds.

**Table S6.** AntiSMASH v.8.0 (Blin et al., 2025) analysis of the *Vreelandella venusta* strain H3 genome; secondary metabolite biosynthetic gene clusters (BGCs) were detected and characterized using antiSMASH with relaxed stringency and compared against the MIBiG database.

**Table S7.** The total number of *Tylos ponticus* Illumina reads obtained for each library ranged from 39,426,338 (Tyl-2H) to 47,463,340 (Tyl-4H).

**Table S8.** CAZymes of *Candidatus* Hepatoplasma of *Tylos ponticus* identified solely through DIAMOND hits against the CAZy database.

**Figure S1.** Alpha rarefaction curves of the microbial communities associated with the hepatopancreas and gut of *Tylos ponticus,* plus the environmental sample. The curves show the number of ASVs at family level as a function of the number of sequencing reads.

**Figure S2.**

The synteny and homology of the ectABC cluster encoding ectoine biosynthetic enzymes (which comprise ectC (ectoine synthase), ectB (diaminobutyrate—2-oxoglutarate transaminase), and ectA (diaminobutyrate acetyltransferase)), with an upstream open reading frame encoding a transcriptional repressor) with those found in *Halomonas hydrothermalis* (NZ_CP023656.1.region003), *Halomonas meridiana* (NZ_CP024621.1.region001), and *Halomonas piezotolerans* (NZ_CP048602.1.region001).

Synteny and homology of the ectABC cluster were visualized using Clinker (Gilchrist and Chooi, 2021) with GenBank files generated by antiSMASH v.8.0 (Blin et al., 2025).

**Figure S3**.

The results of the metagenomic profiling from the hepatopancreas (midgut caeca) of *Tylos ponticus*, specimens Tyl-2H (top) and Tyl-4H (bottom), visualized as a pie chart using Krona.
