# Supplementary material for: Understanding Hepatopancreas-Associated Microbiota in the Supralittoral Tylos ponticus (Crustacea, Isopoda, Oniscidea): Insights from Next-Generation Sequencing Approaches
Source: Microb Ecol. 2026 May 23;89:125. doi: 10.1007/s00248-026-02785-4 (PMC13264560; doi:10.1007/s00248-026-02785-4)
Supplement: Supplementary file 2 [file 248_2026_2785_MOESM4_ESM.docx]

**Table S7**

Filename	Tyl-2H

Encoding	Sanger / Illumina 1.9

Total Sequences	47.463.34 0paired-end (PE)

Sequences flagged as poor quality	0

Sequence length	150

%GC	25% - 45%

Filename	Tyl-4H

Encoding	Sanger /	Illumina 1.9

Total Sequences	39.426.338 paired-end (PE)

Sequences flagged as poor quality	0

Sequence length	150

%GC	36%
