# Supplementary material for: Understanding Hepatopancreas-Associated Microbiota in the Supralittoral Tylos ponticus (Crustacea, Isopoda, Oniscidea): Insights from Next-Generation Sequencing Approaches
Source: Microb Ecol. 2026 May 23;89:125. doi: 10.1007/s00248-026-02785-4 (PMC13264560; doi:10.1007/s00248-026-02785-4)
Supplement: Supplementary file 3 [file 248_2026_2785_MOESM5_ESM.docx]

**Table S5**

Gene ID	EC#	HMMER	dbCAN_sub	DIAMOND	Signal peptide	#ofTools

BBBOOGGN_00142	-	-	-	GT119	N	1

BBBOOGGN_00179	-	-	-	CBM48

+GH13_8	N	1

BBBOOGGN_00193	-	-	-	CE4	N	1

BBBOOGGN_00211	-	-	-	GH1	N	1

BBBOOGGN_00266	-	-	-	GH28	N	1

BBBOOGGN_00270	-	-	-	GT1	N	1

BBBOOGGN_00315	-	-	-	GH13_31	N	1

BBBOOGGN_00424	-	-	-	GT2	N	1

HMMER: dbCAN (E-Value < 1e-15, coverage > 0.35)  DIAMOND: CAZy (E-Value < 1e-102)  HMMER: dbCAN-sub (E-Value < 1e-15, coverage > 0.35)
