# Supplementary figures and images for: Understanding Hepatopancreas-Associated Microbiota in the Supralittoral Tylos ponticus (Crustacea, Isopoda, Oniscidea): Insights from Next-Generation Sequencing Approaches
Source: Microb Ecol. 2026 May 23;89(1):125. doi: 10.1007/s00248-026-02785-4 (PMC13264560; doi:10.1007/s00248-026-02785-4)

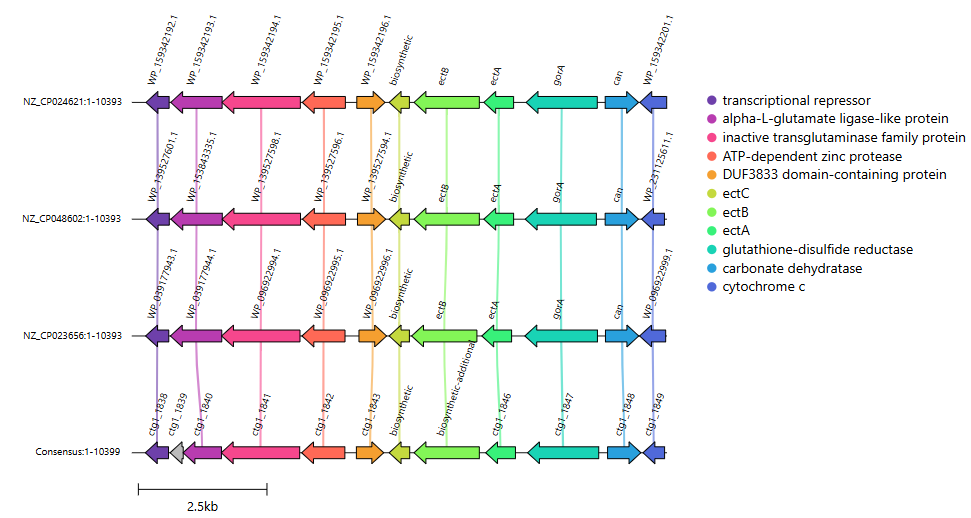

Supplement: Supplementary file 2 — Supplementary Material 2 (PNG97.4 KB) [file 248_2026_2785_MOESM2_ESM.png]

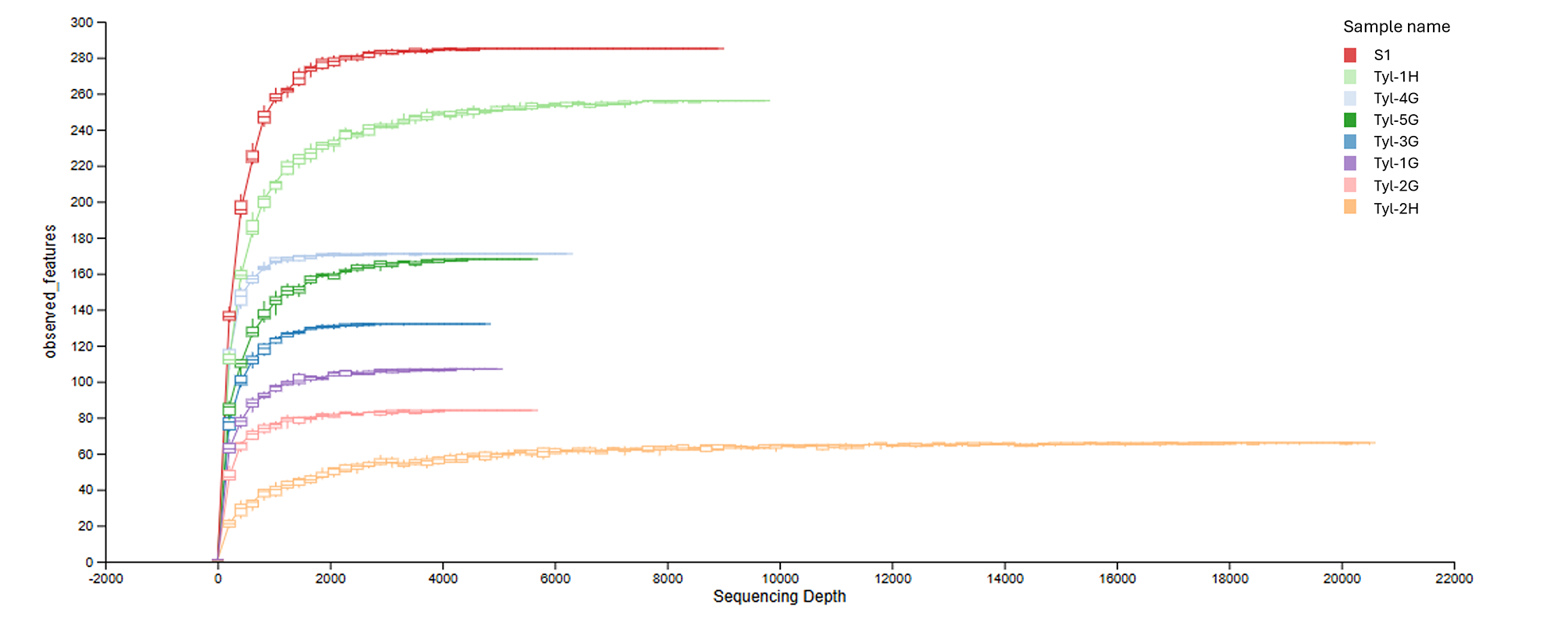

Supplement: Supplementary file 8 — (PNG 170 KB) [file 248_2026_2785_Fig11_ESM.png]

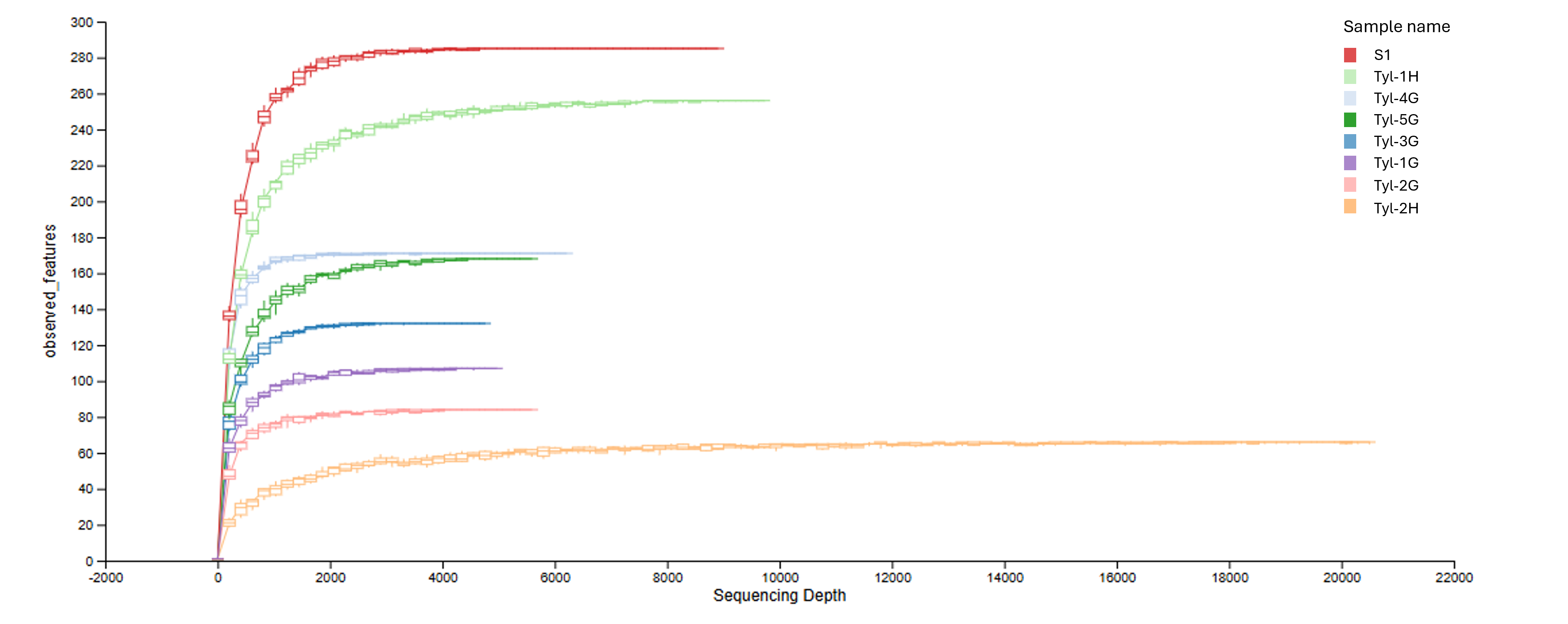

Supplement: Supplementary file 9 — High Resolution Image (TIF 1.05 MB) [file 248_2026_2785_MOESM8_ESM.tif]

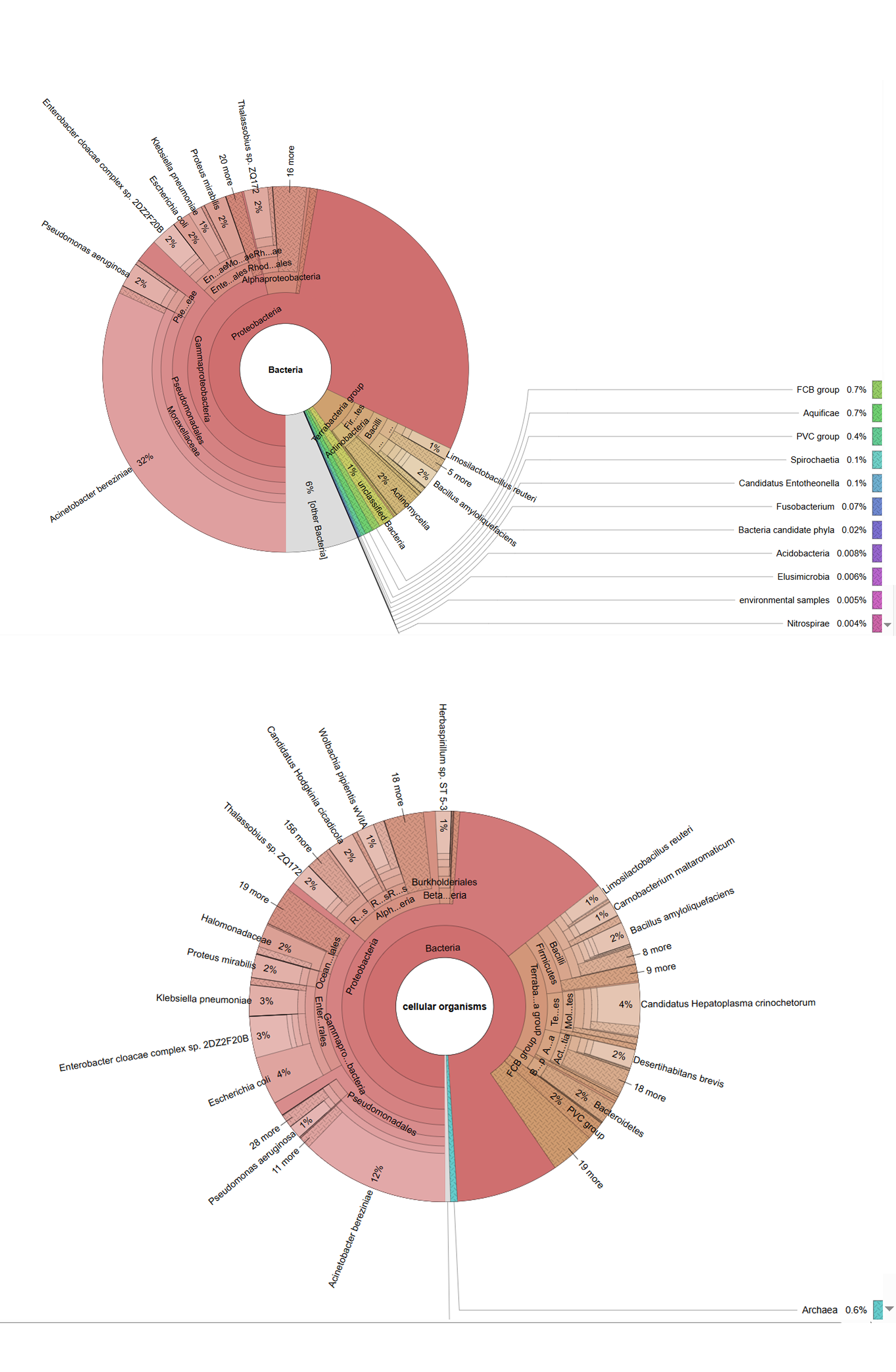

Supplement: Supplementary file 12 — (PNG 1.36 MB) [file 248_2026_2785_Fig12_ESM.png]

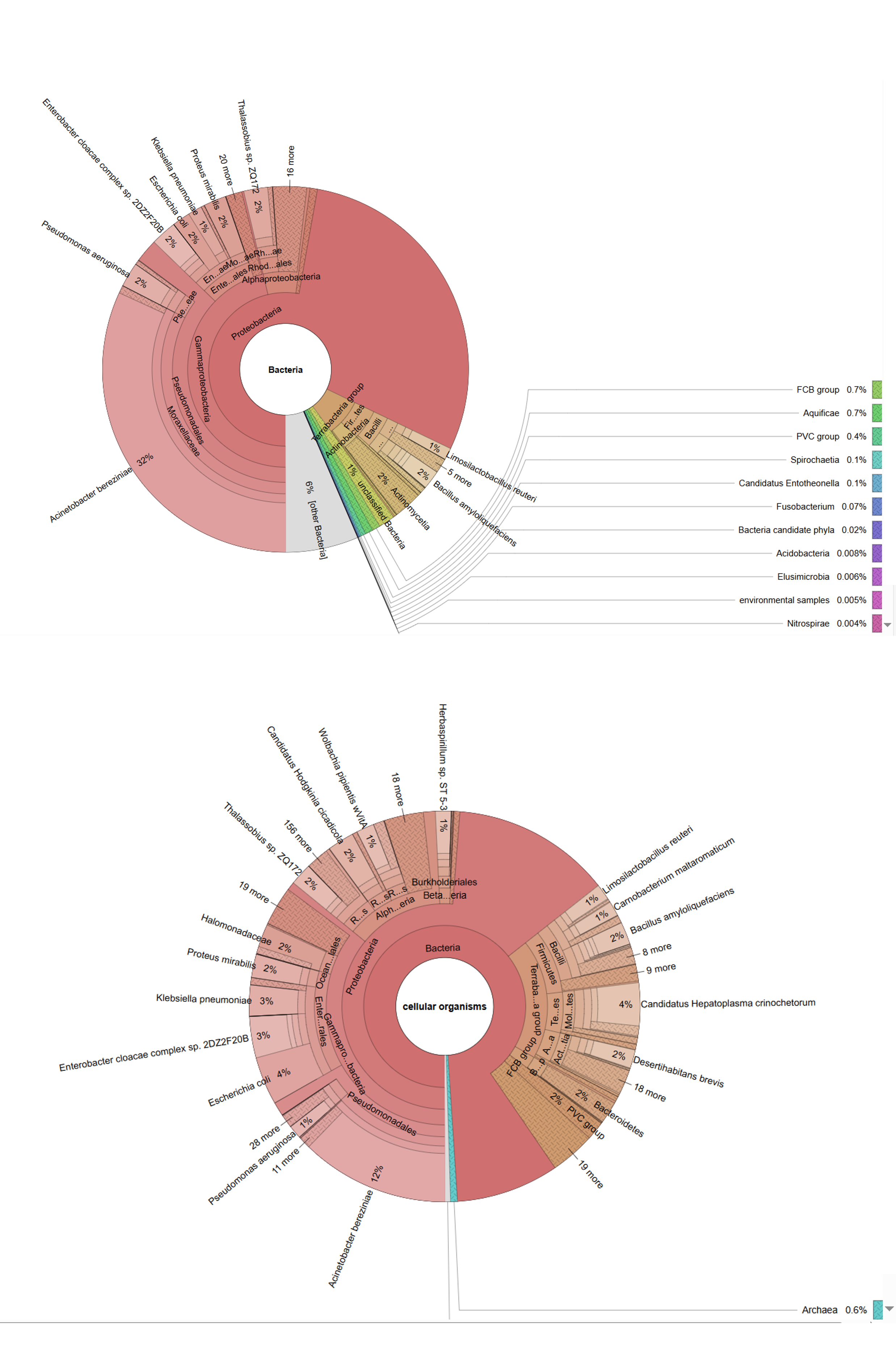

Supplement: Supplementary file 13 — High Resolution Image (TIF 3.15 MB) [file 248_2026_2785_MOESM11_ESM.tif]
