# Supplementary material for: Understanding Hepatopancreas-Associated Microbiota in the Supralittoral Tylos ponticus (Crustacea, Isopoda, Oniscidea): Insights from Next-Generation Sequencing Approaches
Source: Microb Ecol. 2026 May 23;89(1):125. doi: 10.1007/s00248-026-02785-4 (PMC13264560; doi:10.1007/s00248-026-02785-4)
Supplement: Supplementary file 4 — Supplementary Material 4 (DOCX 13.9 KB) [file 248_2026_2785_MOESM4_ESM.docx]

**Table S7**

Filename Tyl-2H

Encoding Sanger / Illumina 1.9

Total Sequences 47.463.34 0paired-end (PE)

Sequences flagged as poor quality 0

Sequence length 150

%GC 25% - 45%

Filename Tyl-4H

Encoding Sanger / Illumina 1.9

Total Sequences 39.426.338 paired-end (PE)

Sequences flagged as poor quality 0

Sequence length 150

%GC 36%
