# Supplementary material for: Understanding Hepatopancreas-Associated Microbiota in the Supralittoral Tylos ponticus (Crustacea, Isopoda, Oniscidea): Insights from Next-Generation Sequencing Approaches
Source: Microb Ecol. 2026 May 23;89(1):125. doi: 10.1007/s00248-026-02785-4 (PMC13264560; doi:10.1007/s00248-026-02785-4)
Supplement: Supplementary file 5 — Supplementary Material 5 (DOCX 14.3 KB) [file 248_2026_2785_MOESM5_ESM.docx]

**Table S5**

Gene ID EC# HMMER dbCAN_sub DIAMOND Signal peptide #ofTools

BBBOOGGN_00142 - - - GT119 N 1

BBBOOGGN_00179 - - - CBM48

+GH13_8 N 1

BBBOOGGN_00193 - - - CE4 N 1

BBBOOGGN_00211 - - - GH1 N 1

BBBOOGGN_00266 - - - GH28 N 1

BBBOOGGN_00270 - - - GT1 N 1

BBBOOGGN_00315 - - - GH13_31 N 1

BBBOOGGN_00424 - - - GT2 N 1

HMMER: dbCAN (E-Value < 1e-15, coverage > 0.35)  DIAMOND: CAZy (E-Value < 1e-102)  HMMER: dbCAN-sub (E-Value < 1e-15, coverage > 0.35)
